# Evaluating the theranostic potential of ferumoxytol when combined with radiotherapy in a mammary dual tumor mouse model

**DOI:** 10.1002/mp.17888

**Published:** 2025-05-21

**Authors:** Deng‐Yuan Chang, Joseph P. Speth, Matthew L. Scarpelli

**Affiliations:** ^1^ School of Health Sciences Purdue University West Lafayette Indiana USA

**Keywords:** ferumoxytol, radiotherapy, tumor associated macrophages

## Abstract

**Background:**

The radiation‐induced abscopal effect (RIAE) is a desirable phenomenon involving radiation‐induced activation of the immune system and regression of metastatic disease after local radiotherapy. However, the majority of patients undergoing radiotherapy do not experience abscopal responses. One potential barrier to the RIAE is tumor‐associated macrophages (TAMs), which can be recruited to the tumor after radiotherapy and have an immunosuppressive effect on the tumor microenvironment (TME).

**Purpose:**

We aim to evaluate the dual capabilities of the FDA‐approved iron nanoparticle ferumoxytol for (1) enhancing the RIAE and (2) measuring TAMs by magnetic resonance imaging (MRI). We hypothesized that (1) the immunomodulating effect of ferumoxytol could enhance the RIAE by repolarizing the M2 TAMs to M1 TAMs, and (2) the TAMs could be non‐invasively imaged by ferumoxytol‐MRI.

**Methods:**

Twenty‐eight BALB/c mice were subcutaneously implanted with 4T1 primary orthotopic tumor (mammary fat pad) and flank tumor (abscopal tumor). At 14 days post‐implantation, mice were separated into four groups: control (Ctrl), radiotherapy (RT) only (8‐Gy×3), ferumoxytol only (FMX; 40 mg/kg) and combined (Comb) group (a single dose of 40 mg/kg FMX 24 h prior to 8‐Gy×3) (*n* = 7 mice per group; 56 tumors). At 23‐ and 24‐day post‐implantation the pre‐ and post‐FMX injection MRI was performed for mice in FMX and Comb group. The percent change in transverse relaxation time (%T2*) from pre to post ferumoxytol injection was calculated from MR images for both tumors and lymph nodes (LNs). At 25 days post‐implantation, both tumors were harvested, and the TAMs were analyzed by flow cytometry.

**Results:**

At 25 days post‐implantation, the primary tumor volume in the RT and Comb groups was significantly lower than the Ctrl and FMX groups (*p *< 0.05). No significant size difference of abscopal tumors was observed among all groups. In addition, there was no significant difference in lung metastasis nodules. A significant decrease in %T2* values of tumors and LNs in the FMX and Comb group 24 h post‐ferumoxytol injection was observed, suggesting ferumoxytol uptake in TAMs. The flow cytometry result showed that the CD80^+^ CD206^−^ M1 macrophage population was similar among all tumors and groups. The CD80^−^ CD206^+^ M2 macrophage population was also similar in all tumors and groups, with the exception of the FMX group, where the M2 tumor macrophage levels were significantly higher when compared to the Ctrl group (*p *< 0.05). Tumors in the FMX group had a significant negative Pearson correlation between tumor %T2* change and M1 tumor macrophage levels (*r* = −0.76, *p *< 0.05) but this correlation was not significant in any other treatment group.

**Conclusions:**

Radiotherapy combined with ferumoxytol led to significant growth delays of irradiated tumors, but no abscopal effects were observed in non‐irradiated tumors. Additionally, our hypothesis that the immunomodulating effect of ferumoxytol could enhance the RIAE by repolarizing the M2 TAMs to M1 was not supported by our findings. However, our second hypothesis that the TAMs could be non‐invasively imaged by ferumoxytol‐MRI was supported by our findings. This includes observation of a significant negative correlation between M1 TAMs and %T2* change in tumors in the ferumoxytol treatment group.

## 1 INTRODUCTION

Radiation therapy is a common procedure for cancer treatment as it is non‐invasive and highly effective for local tumor control. Radiation causes DNA damage, leading to cell cycle arrest or tumor cell death.^1^ In addition, immunogenic cell death can be caused by radiation. This phenomenon includes release of tumor‐associated antigens (TAAs) and boosts the immune system against the tumor cells, including metastatic tumors.^2^ This “off‐target” phenomenon, wherein local radiotherapy leads to regression of distant metastatic disease, is called the “abscopal effect,” and it was first published by Dr Mole in 1953.^3^

The radiation‐induced abscopal effect (RIAE) has several potential advantages, including regression of tumors outside the primary radiation field, reducing the toxicity of therapy, and lowering the probability of tumor recurrence. Several studies have observed evidence of RIAE.^1^, ^4^, ^5^, ^6^ However, this phenomenon is rare in the clinic. The rareness of RIAE could be at least partly caused by radiation‐induced immunosuppression, which has been shown to depend on the radiation dose and the delivery method.^1^, ^7^, ^8^ One reason for radiation‐induced immunosuppression is the relative increase in the number of suppressive cells in the tumor microenvironment (TME), such as tumor‐associated macrophages (TAMs).^9^ TAMs in conjunction with tumor cells release immunosuppressive cytokines, leading to a suppressive TME.^10^, ^11^ In addition, immunosuppressive TAMs are abundant and recruited to the TME following radiation.^10^, ^12^ These properties make TAMs crucial to the therapeutic outcome of cancer patients who are treated with radiotherapy.

Several strategies have been used to reduce the radiation‐induced immunosuppressive effect and enhance the RIAE. For example, hypo‐fractionated radiotherapy has been observed to have immune‐boosting effects and reduce normal tissue toxicity with remarkable tumor control.^13^ The radiation delivered in this method is in a moderate dose range (3–10 Gy/fx), which promotes MHC class I molecule expression on the tumor cells, facilitates the release of TAAs, and activates the CD8^+^ T cell anti‐tumoral response against the primary tumor or even metastases.^13^ Another strategy to reduce radiation‐induced immunosuppression involves targeting TAMs. Several therapies, such as granulocyte‐macrophage colony‐stimulating factor (GM‐CSF) and anti‐CSF‐1 antibody, have been used to reprogram the recruited monocytes to differentiate into anti‐tumoral M1 macrophages instead of tumor‐supportive M2 macrophages.^10^, ^14^, ^15^ In addition, blockage of CD47, a “Don't eat me” signaling molecule expressed on some cancer types, combined with radiotherapy has shown to activate the phagocytosis ability of macrophages to enhance the abscopal effect in a non‐small‐cell lung cancer (NSCLC) model.^16^ With the help of these strategies, more abscopal effects have been observed and some cancer types considered as immune suppressive, or an immune desert, have become more responsive.^17^, ^18^ However, despite these promising studies, not all patients experience the abscopal effect, even with these immune‐boosting strategies, warranting further investigation of RIAEs and strategies to enhance them.

Ferumoxytol is one probe for investigating TAMs. It is an FDA approved iron oxide nanoparticle coated with carboxymethyl‐dextran and it is taken up by macrophages in the reticuloendothelial system (RES) in the days following administration.^19^ While FDA approved for treating iron deficiency, ferumoxytol is also used off‐label as an MRI contrast agent. The iron ions of ferumoxytol shorten both the T1 and T2 relaxation times, showing brighter signal on the T1 images or darker signal on the T2 images, respectively. This has been studied for patients with breast, prostate, and brain cancers.^20^, ^21^, ^22^ Surprisingly, several studies have found that ferumoxytol has an immunomodulating effect. For example, it can polarize the immunosuppressive M2 TAMs into anti‐tumoral M1 TAMs,^23^, ^24^ and it can promote differentiation of myeloid derived suppressive cells (MDSCs) into macrophages.^25^ These findings make it potentially suitable to be repurposed as an immunomodulating agent to improve the immunosuppressive TME while also being used as a non‐invasive tool to monitor TAMs via MRI.

In this study, two main hypotheses were formulated to determine the potential of combining ferumoxytol with radiotherapy for improving the TME: (1) ferumoxytol will reduce the immunosuppressive TME and enhance RIAEs by polarizing the M2 TAMs into M1 TAMs, and (2) the ferumoxytol‐induced signal changes on the MR images can be a non‐invasive indicator of macrophages. To test these hypotheses, we utilized the 4T1 dual tumor mouse model treated with a combination of ferumoxytol and hypo‐fractionated radiotherapy. The 4T1 mammary tumor was utilized as it is a model of triple‐negative breast cancer (TNBC). Breast cancer and in particular TNBCs, have relatively high levels of TAMs.^10^, ^26^ In addition, the overall survival of TNBC patients after radiotherapy is significantly lower compared to patients with other subtypes after radiation therapy, suggesting further improvements in treatment are needed.^27^

## 2 MATERIALS AND METHODS

### 2.1 Animals

Eight to ten‐week‐old female BALB/c mice were purchased from Envigo (Lafayette, Indiana) and bred in our animal facilities under specific pathogen‐free conditions. All animal experiments were approved by the Institutional Animal Care and Use Committee of Purdue University.

### 2.2 Cell culture

A mouse triple‐negative mammary cancer cell line, 4T1, and a mouse monocyte/macrophage cell line, Raw 264.7, were used in this study. Both 4T1 and Raw 264.7 were purchased from ATCC. Cryopreservation of large quantities of low passages (below 5) was performed, and cells with passage numbers below 30 were used in this study. Cells were routinely cultured in RPMI‐1640 medium supplemented with 10% FBS and 1% penicillin/streptomycin (4T1) or DMEM medium supplemented with 10% FBS and 1% penicillin/streptomycin (Raw 264.7). All cell lines were maintained at 37°C in a humidified 5% CO_2_/95% air incubator.

### 2.3 Reagents

In general, the clones of antibodies we used were commercially available through reputable vendors with multiple cited publications confirming prior use for flow cytometry. The following antibodies were used: PE‐hamster anti mouse CD11c (BD, clone N418), Alexa flour 700‐rat anti mouse CD11b (BD, clone M1/70), PE‐CF594‐rat anti mouse F4/80 (BD, clone T45‐2342), APC‐ rat anti mouse CD206 (eBioscience, clone MR6F3), V450‐rat anti mouse CD45 (BD, clone 30‐F11), FITC‐rat anti mouse I‐A/I‐E (MHC II), (BD, clone 2G9), APC‐rat anti mouse CD80 (Southern Biotech, clone 1G10), and PE‐rat anti mouse CD206 (BD, clone Y17‐505). The specific fluorophores were chosen to avoid the photon spectrum emissions overlapping within each detector's bandwidth on our flow cytometer. CD80 and CD206, were chosen as TAM markers based on previous studies, including similar work published by Zanganeh et al.^23^ The following reagents were used, Ferumoxytol (Feraheme, AMAG Pharmaceuticals), SYTOX Blue Nucleic Acid Stain (Invitrogen), 10× RBC lysis buffer (Invitrogen), Collagenase A (Roche, Basel, Switzerland), DNase I (STEMCELL Technologies), EDTA (Thermo Scientific), BSA (Thermo Scientific), FcR binding inhibitor (eBioscience, USA), poly I:C (Invitrogen), Cell Counting kit 8 (Abcam), HMGB1 ELISA kit (Novus Biologicals), and TNF‐α ELISA kit (Invitrogen).

### 2.4 Therapeutic study in 4T1 dual tumor model

**FIGURE 1:**
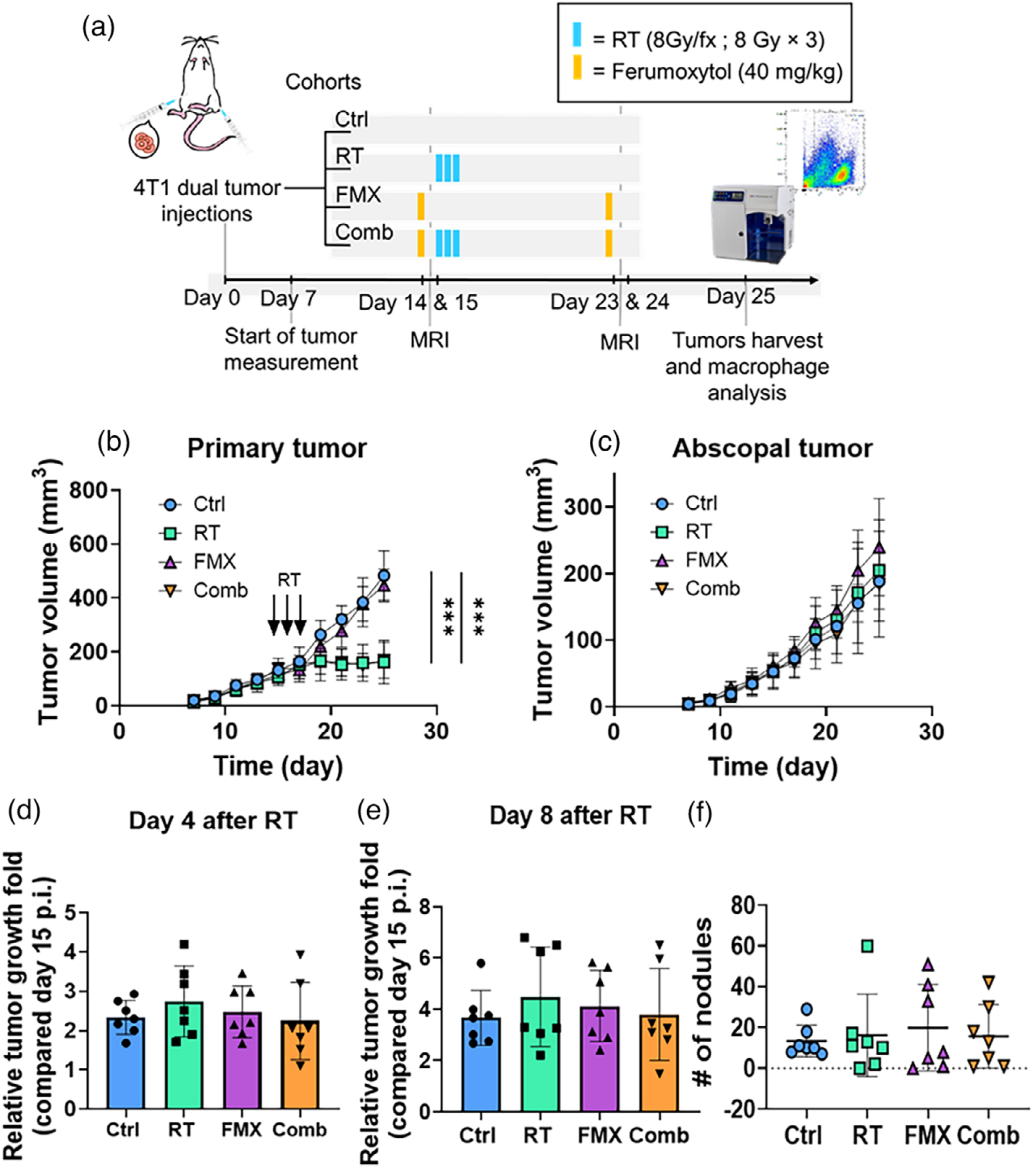
Ferumoxytol could not enhance both local and abscopal tumors inhibition following irradiation of 4T1 tumors. (a) Schematics depicting the therapeutic schedule for RT and ferumoxytol administered in 4T1 dual tumor mouse model. (b) Growth curves of orthotopic 4T1 primary tumors in BALB/c mice with the indicated treatments (*n* = 7 per group). The black arrow indicates the radiation treatment in three consecutive days (8 Gy × 3). The vertical bars with asterisks indicate the RT and Comb groups had significantly reduced tumor volume relative to the Ctrl group at Day 25. (c) Growth curves of 4T1 abscopal tumors in BALB/c mice (*n* = 7 per group). In this case there were no significant differences between any of the other treatment groups and the Ctrl group. (d) and (e) Relative growth fold of flank tumor volume at (d) 4‐day and (e) 8‐day post‐irradiation. No significant differences were noted between treatment groups. (f) Total number of lung metastatic nodules were counted among all treatment groups. No significant differences were noted across treatment groups. Data are present in mean ± SD. Tumor growth over time was compared by two‐way ANOVA with Tukey's correction. (d)–(f) were compared by unpaired Student's *t*‐test with Mann–Whitney test. *p < 0.05; **p < 0.01; ***p < 0.001; ****p < 0.0001. Comb, combined ferumoxytol with radiotherapy; Ctrl, control; FMX, ferumoxytol; RT, radiotherapy.

For the dual tumor 4T1 mouse model, female BALB/c mice were subcutaneously injected with single cell suspension of 1 × 10^5^ 4T1 tumor cells in 50 µL of PBS in the 4th mammary fat pad (as the primary tumor or irradiated tumor) at the right side, and 5 × 10^4^ 4T1 tumor cells in 50 µL of PBS in the upper left thigh (as the flank tumor or abscopal tumor) at the left side on Day 0. The therapy schedules are shown in Figure 1a. Briefly, after 7‐day post‐implantation, the size of tumors was measured every other day using a caliper (Fisherbrand) and the tumor volume was calculated as 4 × π × length (mm) × width (mm) × height (mm)/3. When the volume of primary tumor was around 100 mm^3^, mice were separated into four groups: Ctrl, radiation only (RT; 8‐Gy × 3), ferumoxytol only (FMX; 40 mg/kg) and Combined group (Comb; 40 mg/kg FMX and 8‐Gy × 3) (*n* = 7 mice per group; 56 tumors). At 14 days post tumor implantation a single dose of 40 mg/kg ferumoxytol was administered intravenously in the FMX and Comb group. Only the primary tumor of the mice in RT and Comb groups received radiation (8‐Gy × 3) on Days 15–17 post tumor implantation. At 23 days post tumor implantation, a second dose of 40 mg/kg ferumoxytol was administered in the FMX and Comb group. At 25 days post tumor implantation, both tumors were harvested, and the TAM population was analyzed.

### 2.5 Hypo‐fractionated radiotherapy

Animal irradiation was performed using an X‐RAD 320 irradiator cabinet (Precision x‐ray, North Brandford, CT, USA). Mice were under anesthesia using 5% isoflurane through a nose cone supplied to the animal stage. The irradiations were performed using an x‐ray energy of 320 kVp and a current of 50 mA with a dose rate of ∼175 cGy/min. These beam settings are the default settings on our X‐RAD 320 machine, and they were utilized to match other preclinical studies, many of which utilize similar cabinet irradiators. The primary beam was filtered by 2 mm Al prior to the animal stage. The rationale for the 2 mm Aluminum is to harden the beam and provide a more uniform distribution throughout the mouse. The beam was collimated to 16 × 16 mm^2^ to cover the whole tumor, and the irradiation spot was localized using a radiochromic film (Thermo Scientific). This choice of field size was selected to optimize tumor coverage, while limiting dose to normal tissues. The rest of mouse body outside the collimation was covered by the lead shield with a thickness of 3 mm.

### 2.6 MRI measurements

During the MRI measurements, the tumor‐bearing mice underwent MRI using a 7‐Tesla Bruker Biospec preclinical scanner and were kept under deep anesthesia (2% isoflurane in oxygen). Baseline MRI measurements (or pre‐injection) were acquired at Day 14 and Day 23 post‐tumor implantation, prior to ferumoxytol injection on these days. Corresponding post‐injection MRI measurements were acquired, 24 h later, at Days 15 and 24 post‐tumor implantation. T_2_‐weighted anatomic Turbo RARE images were acquired with a slice thickness of 1 mm, using 256 × 256 pixels of 0.117 mm each, 20 slices, TR = 1582.99 ms, TE = 24 ms and nex = 4. Scans for T2* mapping were acquired using a multi‐gradient echo sequence with a slice thickness of 1 mm, using 128 × 128 pixels of 0.234 × 0.234 mm, 20 slices, TR = 1500 ms, nex = 2, and echo times of 2, 6, 10, 14, 18, 22, 26, 30, 34, and 38 ms. The MR data were processed with 3D Slicer and MATLAB. ROIs that contained mammary tumor, flank tumor, and left and right inguinal LN were drawn in each slice of the MR images. Voxel‐by‐voxel T2* relaxation times of these organs were acquired by fitting the mono‐exponential equation from the signal intensity at each echo using a MATLAB script developed in‐house. The mean T2* voxel values and the volume of organs and tumors were obtained. Then, the ΔT2* of the tumors were calculated by subtracting the pre‐injection mean transverse relaxation rate (time), $T2_{\mathrm{pre}}^{*}$, from the post‐injection mean transverse relaxation rate (time), $T2_{\mathrm{post}}^{*}$. The ΔT2* can be obtained from the first pre‐ and post‐FMX injection MR images before radiotherapy (on Days 14 and 15), and also from the second set of MR images after the radiotherapy (on Days 23 and 24). Next, the %T2* values of tumors and LNs was calculated by Equation 1, as shown below:

(1) $$\%\;T2^{*}=\frac{\Delta T2^{*}\times 100\%}{T2_{\mathrm{pre}}^{*}}\;$$

### 2.7 Bouin's fixative staining

Mouse lung tissue was harvested and fixed in Bouin's fixative buffer (Sigma Diagnostics) for 24 h and then soaked in the anhydrous ethanol to restore the color of lung tissue. The white nodules of metastatic breast cancer on pulmonary surface were observed and counted.

### 2.8 Tissue dissociation and flow cytometry

Mice were euthanized with a cervical dislocation under anesthesia (isoflurane). Then, the transcardial perfusions were conducted with 10 mL of ice‐cold PBS. To create tumor cell suspensions, both tumors were harvested, placed into ice‐cold PBS, minced with a razor blade, washed and incubated for 30–40 min at 37°C in DMEM (Corning) with 2.0 mg/mL Collagenase A (Roche) and 50 units/mL DNase I (STEMCELL Technologies). Single‐cell suspensions were prepared by filtering through 40‐µm nylon strainers (Fisherbrand). Cells were washed, then red blood cells were lysed by ACK‐lysis buffer (Invitrogen eBioscience) for 3 min on ice, and cells were washed again in PBS containing 2.0 mM EDTA and 0.5% BSA (FACS buffer). Thereafter, 10^6^ cells in 50 µL of FACS buffer were incubated for 15 min on ice with 5 µL of Fc Receptor Binding Inhibitor (eBioscience, USA) to reduce non‐specific binding. Cells were then incubated for 30 min on ice with fluorescently labeled primary monoclonal antibodies. The following antibodies were used for analyzing the phenotype of macrophages: anti‐CD45‐V450, anti‐CD11b‐Alexa Flour 700, anti‐F4/80‐PE CF594, anti‐CD80‐APC, anti‐CD206‐PE, anti‐MHC II‐FITC, anti‐CD11c‐PE, and anti‐CD 206‐APC. Sytox blue was used to exclude the dead cells. Acquisition was performed on an LSRII Fortessa flow cytometer (BD Biosciences, New Jersey, USA). Data analysis was performed using FlowJo Version 10.10.0 (BD Biosciences, New Jersey, USA). Live singlets were gated using FSC‐A/FSC‐H. Gates were drawn using fluorescent minus one control tubes.

### 2.9 Cytokine analysis

To examine cytokines in mouse plasma, blood was collected at Days 13, 19, and 25 post‐tumor implantation into EDTA‐coated tubes and centrifuge twice (1st, 3000 rpm; 2nd 4000 rpm) to obtain plasma. The level of inflammatory factors HMGB1 and TNF‐α were measured with corresponding ELISA kits, including mouse HMGB1 ELISA kit and mouse TNF‐α ELISA kit, according to the manufacturer's instructions.

### 2.10 Statistics

All results show mean values ± standard deviation unless otherwise noted. Statistical analyses were performed using GraphPad Prism 10 (La Jolla CA, USA, www.graphpad.com). Significance between mean values of different treatment groups was analyzed using Two‐way ANOVA and/or unpaired Student's *t*‐test. These statistical tests were chosen as they provide a reliable estimate of significant differences between groups and are relatively easy to interpret. Additional rationale is that these statistical tests are commonly used in similar preclinical studies making it easier to compare results across studies. *p*‐Values less than 0.05 were considered statistically significant.

## 3 RESULTS

### 3.1 Effect of ferumoxytol and combined RT on the RIAE

We first evaluated the ferumoxytol and radiation‐induced bystander effect in vitro with the 4T1 tumor cells co‐cultured with the Raw 264.7 cells. This showed tumor proliferation can be inhibited if macrophages are polarized into an M1 phenotype (Figure S1). In vivo, in our dual tumor model, we observed significantly delayed growth of the primary, irradiated, tumors in the RT and Comb groups relative to the Ctrl group. However, this growth delay was not observed in the abscopal, non‐irradiated, tumors (Figure 1b,c and Figure S2). The fold change in the abscopal tumor volume at 4 days post‐irradiation or 8 days post‐irradiation relative to the volume before the radiation treatment is shown in Figure 1d and e, respectively. The tumor volume fold changes at 4 or 8 days post‐irradiation was not significantly different across groups, suggesting these treatments (RT, FMX, and Comb) do not induce measurable abscopal effects compared to the Ctrl group. These treatments showed no systemic toxicity effect evaluated as the percent change of body weight (Figure S3A) but did show significantly increased spleen weight in the Ctrl and FMX group relative to the RT and Comb groups (Figure S3B). In addition, the immunostimulatory DAMP, HMGB1, was lower in the RT and Comb group compared to the Ctrl and FMX group, albeit the difference was not significant (Figure S3C). The TNF‐alpha level in the RT and FMX groups gradually increased compared to the Ctrl group during the experiment, while in the Comb group, TNF‐alpha peaked at Day 19 and then decreased to a level similar to the Ctrl group (Figure S3D). A positive Pearson correlation was found between the HMGB1 level and the primary tumor size (*p *< 0.0001) or the flank tumor size (*p *= 0.0005) (Figure S3E and S3F).

We also measured the lung metastasis nodules 8 days post‐irradiation (Figure 1f), but no difference in number of pulmonary nodules was observed among all groups. Interestingly, we found the number of lung metastasis was negatively correlated with the %MHC class II^+^ tumor cells in the flank tumors of all groups (Figure S4B, *p *= 0.01), however, this trend was not significant for primary tumors (Figure S4A, *p *= 0.15).

### 3.2 Effect of ferumoxytol and combined RT on tumor T2* values as evaluated by ferumoxytol MRI

**FIGURE 2:**
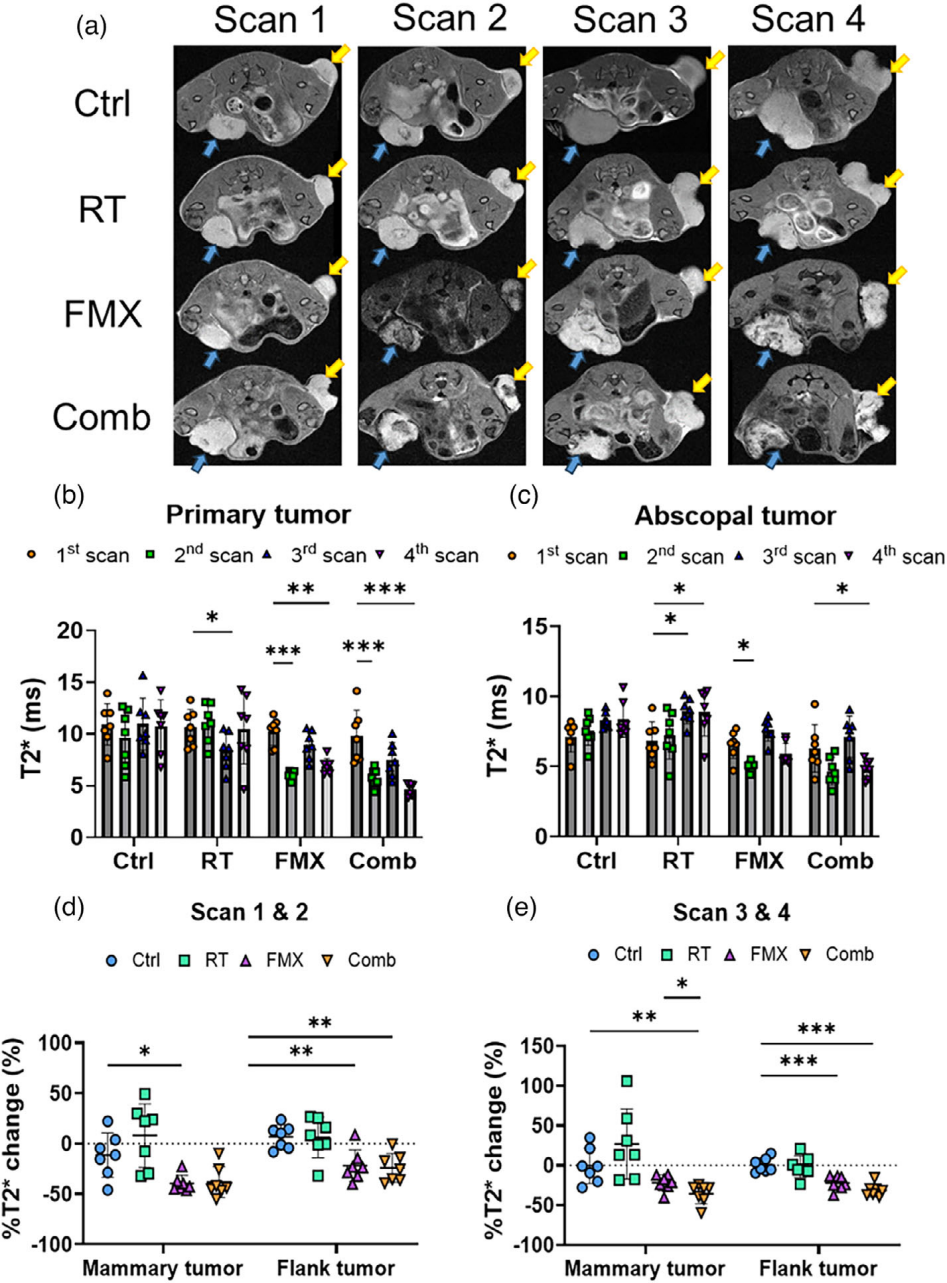
Non‐invasive ferumoxytol MRI evaluation. (a) T2‐weighted MR images (TurboRARE) were taken to visualize the ferumoxytol penetration in both tumors. The blue arrows indicate the primary tumor, and the yellow arrows indicate the abscopal tumor. The dark region in tumors suggests the ferumoxytol penetrated into the tumors. (b) The mean T2* values of primary tumor among all groups in all scans. (c) The mean T2* values of secondary (abscopal) tumor among all groups in all scans. (d) The mean %T2* change of mammary tumor and flank tumor from 1st and 2nd scans in both tumors among all groups at Days 14 and 15 (before the radiation treatment). (e) The mean %T2* change of mammary tumor and flank tumor from 3rd and 4th scans in both tumors among all groups at Days 23 and 24 (6‐day post‐irradiation). (b)–(e) Data were compared by unpaired Student's *t*‐test with horizontal bars representing significant differences between two groups. Data are represented as mean ± SD and are representative of 7 independent experiments. *p < 0.05; **p < 0.01; ***p < 0.001; ****p < 0.0001. Comb, combined ferumoxytol with radiotherapy; Ctrl, control; FMX, ferumoxytol; RT, radiotherapy.

MR images were used to evaluate ferumoxytol penetration in both tumors as well as the size of both tumors and inguinal LNs (Figure 2a). The mean and median T2* values of several organs, and the ΔT2* of tumors at each scan set were summarized (Table S1 and S2 and Figure S5A–S5C). Visually, both the primary and abscopal tumors have reduced signal intensity on the 24 h post‐injection T2‐weighted MR images in the FMX and Comb group compared to Ctrl and RT group. The T2* values of primary tumors significantly decreased from pre‐ to post‐ferumoxytol injection in the FMX and Comb group before and after radiotherapy (Figure 2b). Similar trends were found in abscopal tumors, albeit the extent of T2* decrease was not as significant as in primary tumors (Figure 2c). Before radiotherapy, the %T2* change of both tumors showed no difference between the FMX and Comb group (Figure 2d). After radiotherapy, the %T2* change of primary tumors in the Comb group was significantly lower compared to the FMX group (*p *= 0.02), but this trend was not observed in the abscopal tumors (Figure 2e).

### 3.3 Effect of ferumoxytol and combined RT on the inguinal LN T2* values

**FIGURE 3:**
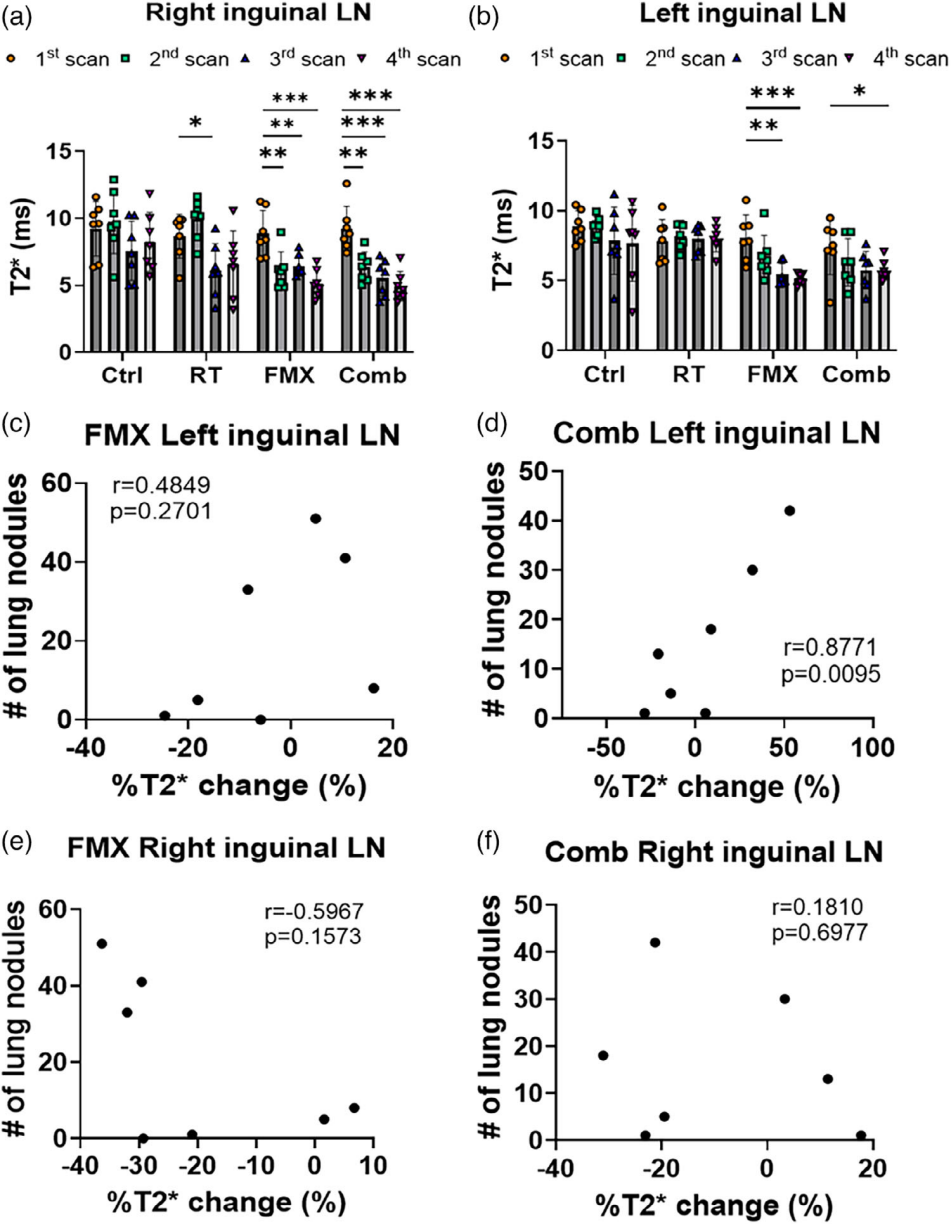
A positive Pearson correlation was observed between %T2* change in inguinal LNs and number of lung nodules. (a) The mean T2* values of right inguinal LN among all groups in all scans. (b) The mean T2* values of left inguinal LN among all groups in all scans. (c)–(d) The Pearson correlation between the mean %T2* change of left inguinal LN and number of lung metastatic nodules in the (c) FMX group and (d) Comb group. (e)–(f) The Pearson correlation between the mean %T2* change of right inguinal LN and number of lung metastatic nodules in the (e) FMX group and (f) Comb group. Data are represented as mean ± SD and are representative of 7 independent experiments. These data were compared by unpaired Student's *t*‐test with horizontal bars representing significant differences between two groups. *p < 0.05; **p < 0.01; ***p < 0.001; ****p < 0.0001. Comb, combined ferumoxytol with radiotherapy; Ctrl, control; FMX, ferumoxytol; LN, Lymph node; RT, radiotherapy.

We investigated the T2* values of inguinal LNs to assess the macrophage infiltration in the LNs. The inguinal LN on the right side is near the radiation field, and the inguinal LN on the left side is near the abscopal tumor, far from the radiation field. The T2* values of inguinal LNs are shown in Figure 3. The T2* values of right LNs in the FMX and Comb group significantly decreased from the first to second MRI scan (pre to post‐ferumoxytol administration). The T2* values further decreased at the 3rd MRI scan in the Comb group but not in the FMX group. At the 4th MRI scan, the T2* values of right LNs in the FMX and Comb group were similar (Figure 3a). The T2* values of left inguinal LNs in the FMX and Comb showed a decrease from the first to second MRI scan (pre to post ferumoxytol administration), and then gradually decreased to plateau at the 4th MRI scan (Figure 3b). In addition, there was a positive Pearson correlation between %T2* change of left inguinal LNs and the number of lung metastatic nodules in the Comb group (*p *= 0.01) but not in the FMX group (*p *= 0.27) (Figure 3c,d). In contrast, no obvious correlation was observed between the %T2* change of right inguinal LNs and the lung metastatic nodules in the FMX group (*p *= 0.16) or Comb group (*p *= 0.70) (Figure 3e,f).

### 3.4 Effect of ferumoxytol and combined RT on immune cell infiltration and the TME

**FIGURE 4:**
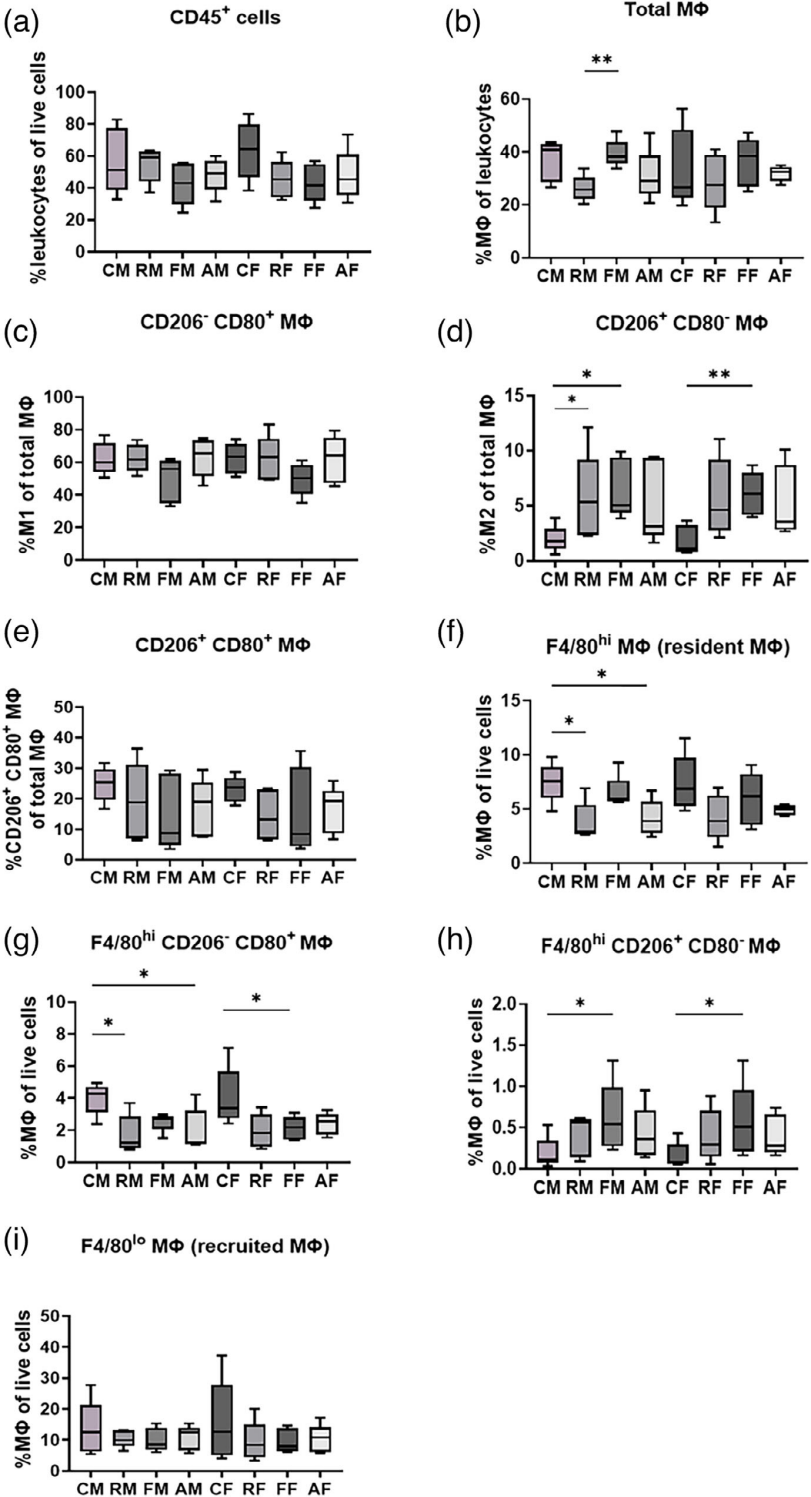
Ferumoxytol combined with radiotherapy did not increase CD80^+^ CD206^−^ M1 tumor macrophage levels. (a) The percentage of leukocytes population (CD45^+^ cells) of total live cells in each tumor (i.e., mammary tumor and flank tumor) among all groups. (b) The percentage of macrophages population of total leukocytes in each tumor among all groups. The percentage of (c) CD80^+^ CD206^−^ M1, and (d) CD80^−^ CD206^+^ M2 population of total macrophage in each tumor among all groups. (e) The percentage of CD80^+^CD206^+^ macrophages. (f) F4/80^hi^ macrophages (resident macrophages). (g) F4/80^hi^ CD206^−^ CD80^+^ M1 (resident) macrophages. (h) F4/80^hi^ CD206^+^ CD80^−^ M2 (resident) macrophages. (i) F4/80^lo^ macrophages (recruited macrophages). Data are represented as the ranges (min–max) of the population and are representative of 5 independent experiments. These data were compared by unpaired Student's *t*‐test with horizontal bars representing significant differences between two groups. *p < 0.05; **p < 0.01; ***p < 0.001; ****p < 0.0001. AF, combined treatment flank tumor; AM, combined treatment mammary tumor; CF, control flank tumor; CM, control mammary tumor; FF, ferumoxytol flank tumor; FM, ferumoxytol mammary tumor; MHC II, major histocompatibility complex class two; MΦ, macrophage; RF, radiotherapy flank tumor; RM, radiotherapy mammary tumor.

Next, we evaluated the immune cell infiltration, such as macrophages and dendritic cells, by flow cytometry to assess how ferumoxytol combined with RT alters the TME. The leukocytes (CD45^+^ cells) in both tumors among all groups were around 50%, from 42.5 ‐ 63.5% of total live cells, with no statistically significant differences among the groups (Figure 4a). Around 30% of leukocytes were macrophages in both tumors among all groups with mean range from 26.1 – 39.4%. The percentage of macrophages was significantly lower in the RT primary tumor compared to the FMX primary tumor. This trend was not significant in corresponding flank tumors (Figure 4b). The dendritic cell (DC) population in both tumors was significantly lower in the RT and Comb group compared to the Ctrl group. This trend was also observed in the FMX group, but it did not reach a statistically significant difference (*p *= 0.06, Figure S6A). Moreover, the MHC class II expressed on the DCs was significantly downregulated in the RT group primary and flank tumors, FMX group flank tumors, and Comb group flank tumors compared to the Ctrl group tumors (Figure S6B). This might suggest these DCs were tolerogenic. The MHC class II expression level on the tumor cells was also investigated. However, around 30% of tumor cells expressed MHC class II (mean range from 26.3 – 37.2%), and there was no difference among groups (Figure S6G). Overall, this suggests ferumoxytol and/or radiotherapy influences the macrophage population but combining the treatments does not lead to obvious improvement in the TME.

### 3.5 Effect of ferumoxytol and combined RT on the classic phenotype of macrophages

To investigate the effect of combined ferumoxytol and RT treatment on the phenotype of macrophages, we evaluated the percentage of several specific macrophage phenotypes within the total macrophage population, including the classic CD80^+^CD206^−^ M1 macrophages and CD80^−^CD206^+^ M2 macrophages. The results showed that the M1 macrophage is the predominant macrophage population in both tumors among all groups. The FMX primary and flank tumors showed lower M1 macrophage levels compared to any other group, although it was not statistically significant (Figure 4c). Interestingly, the FMX primary and flank tumors showed significantly higher levels of M2 macrophages (*p* < 0.05), and this trend was also observed in RT and Comb groups (Figure 4d). The RT, FMX, and Comb groups showed slightly decreased CD80^+^CD206^+^ macrophage levels compared to the Ctrl group but it was not statistically significant (Figure 4e). The mixed M1/M2‐type phenotype (CD80^+^CD206^+^ macrophage) can be considered M1‐to‐M2 or M2‐to‐M1 switching cells, showing high plasticity and a repolarizing effect after treatment. To attempt addressing this ambiguity, we evaluated the median fluorescence intensity (MFI) of CD80, CD206 and MHC II of these biomarkers expressed on the different phenotypes of total macrophages (Figure S7). As determined by flow cytometry, the expression level of CD80 was similar on the CD206^−^CD80^+^ M1 and CD80^+^CD206^+^ mixed phenotypes of macrophages in all tumors among all groups (Figure S7A and S7B). Similarly, the expression level of CD206 was also similar on the CD206^+^CD80^−^ M2 and CD80^+^CD206^+^ mixed phenotypes of macrophages in all tumors among all groups (Figure S7C and S7D). However, the MHC class II expression was significantly lower in the Comb groups on M1, M2 and mixed phenotypes compared to the Ctrl group. In addition, the reduced MHC class II expression on the mixed phenotype was also observed in both tumors of RT and FMX groups (Figure S7E–S7G). On the Contrary, the MHC class II expression increased on the CD80^−^CD206^−^ macrophages in the RT, FMX, and Comb flank tumors (Figure S7H).

To further assess macrophage subtypes, we differentiated the macrophages as resident macrophages or recruited macrophages (i.e., monocytes) by the biomarkers F4/80^hi^ and F4/80^lo^, respectively. The resident macrophage population was significantly lower in the RT and Comb group primary tumors relative to the Ctrl group tumors (Figure 4f). This trend was due to fewer resident F4/80^hi^ M1 macrophages in these groups (Figure 4g). The resident F4/80^hi^ M2 macrophage population was significantly higher in both FMX group mammary and flank tumors compared to the Ctrl group tumors (Figure 4h).

Other subtypes of macrophages, such as resident macrophage (CD45^+^CD11b^+^F4/80^hi^ cells, Figure S6C), recruited macrophages or monocytes (CD45^+^CD11b^+^F4/80^lo^ cells, Figure S8C), and pro‐inflammatory monocytes (CD45^+^CD11b^+^F4/80^lo^MHC II^+^ cells, Figure S6E) were assessed. A subset of mammary resident macrophages, F4/80^hi^ CD206^+^ MHC II^+^ MΦ,^28^ significantly decreased among both tumors in the RT, FMX, and Comb groups (Figure S8A), and this decreased population mainly expressed CD80 (Figure S8B). In addition, the F4/80^hi^MHC II^+^CD80^+^ macrophage population significantly decreased in Comb both tumors (Figure S6D) but the F4/80^lo^MHC II^+^CD80^+^ recruited macrophage population showed no difference among all groups (Figure S6F). No significant difference was observed in other recruited macrophages among all groups (Figures 4i and S8C—S8E).

### 3.6 Correlation between MRI changes and CD206^−^CD80^+^ M1 or CD206^+^CD80^−^ M2 macrophages

**FIGURE 5:**
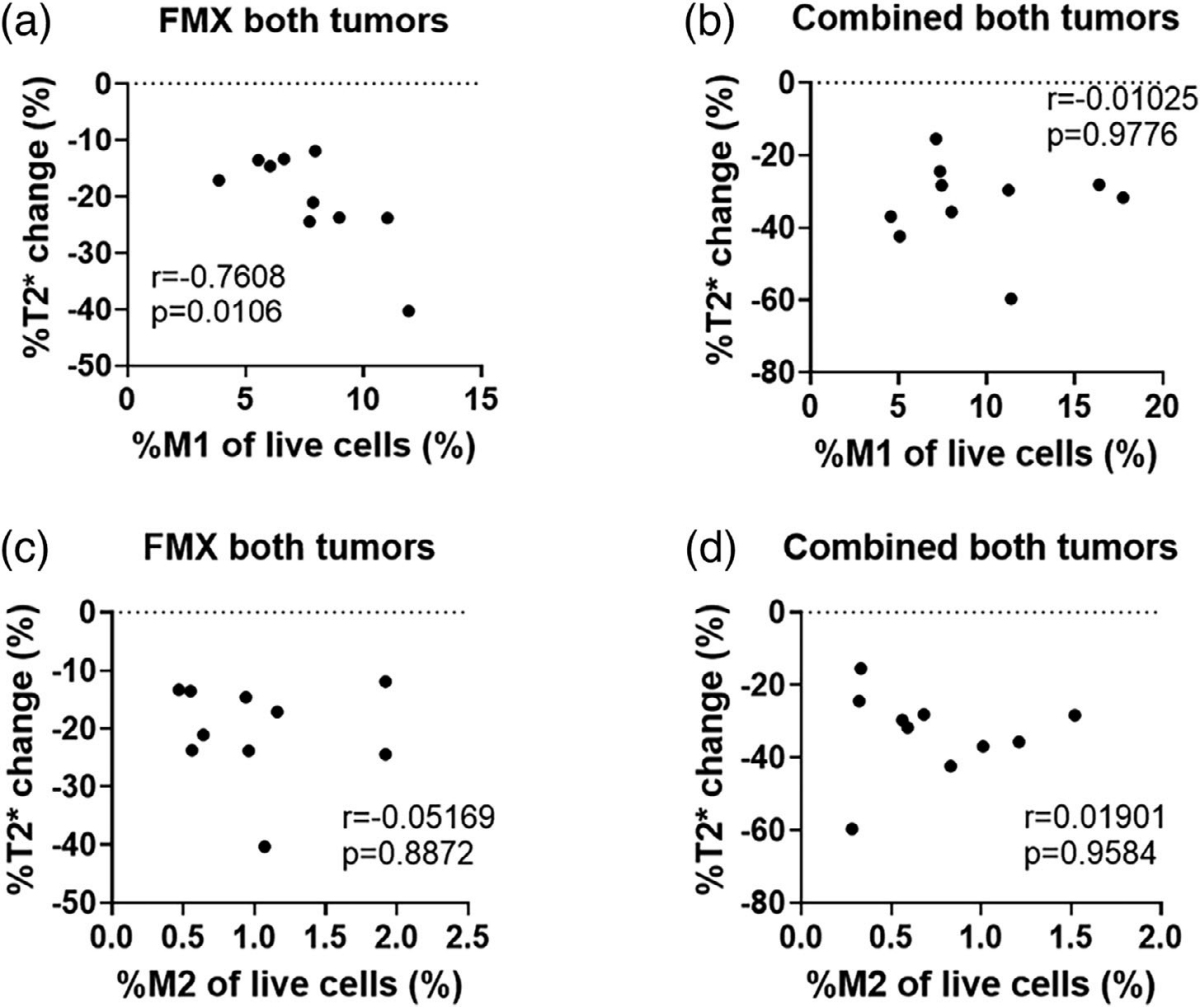
A negative Pearson correlation between %T2* change (from MRI scans on Days 23 and 24) and CD80^+^ CD206^−^ M1 population was observed for tumors of FMX group but not in Comb group. (a)–(b) The Pearson correlation between %T2* change and CD80^+^ CD206^−^ M1 population in (a) FMX group tumors or (b) Comb group tumors. (c)–(d) The Pearson correlation between %T2*change and CD80^−^ CD206^+^ M2 population in (c) FMX group tumors or (d) Comb group tumors. Comb, combined ferumoxytol with radiotherapy; FMX, ferumoxytol; MΦ, macrophage.

To assess if the ferumoxytol signal on MRI can be an indicator of macrophages, the %T2* changes were correlated with the population of macrophage subtypes, as a percentage of total cells. There was a negative correlation between %T2* change and CD206^−^CD80^+^ M1 macrophage population in FMX mammary (*p *= 0.14, Figure S9A) and FMX flank tumors (*p *= 0.10, Figure S9B), which reached statistical significance when the primary and flank tumor data were pooled (*p *= 0.01, Figure 5a). However, in the Comb group, after radiotherapy, there was no correlation among primary (*p *= 0.99, Figure S9C), flank (*p *= 0.96, Figure S9D) or when pooling all tumors together (*p *= 0.98, Figure 5b). There was also no significant correlation between the %T2* change and CD206^+^CD80^−^ M2 population in the FMX group for either the primary tumor (*p *= 0.50, Figure S10A), flank tumor (*p *= 0.11, Figure S10B) or when pooling all tumors (p = 0.89, Figure 5C). Similarly, no significant correlation was observed in the Comb group (*p *= 0.96, Figure 5d and S10C and S10D). No significant correlation between tumor size and %T2* change was observed among both tumors in the FMX and Comb groups (Figure S11A–S11D).

The Pearson correlation was also used to evaluate the relationship between the macrophage population and MRI‐defined tumor size measured 24 days post tumor implantation. A significant negative correlation was found in the FMX group between %M1 population with mammary tumor size (*p *= 0.04, Figure S12A) but not in the flank tumor size (*p *= 0.68, Figure S12B). No significant correlation in the Comb group was found between the %M1 population and tumor size for either flank or mammary tumor (data not shown). No significant correlation was found in the FMX group between the %M2 population and tumor size for either flank or mammary tumor (Figures S12C and S12D and S12G). A positive correlation for the Comb group was found between the %M2 macrophages and the mammary tumor (*p *= 0.15, Figure S12E) and flank tumor (*p *= 0.07, Figure S12F) size. When pooling the Comb group tumors, this correlation between %M2 macrophages and tumor size became statistically significant (*p *= 0.01, Figure S12H). No significant correlation between the population of macrophages and the tumor size was observed in the Ctrl and RT groups (data not shown). Taken together these results suggest the population of M1 macrophages might play a role in the FMX group tumor growth, while the M2 population might be more critical for the growth of Comb group tumors.

## 4 DISCUSSION

TAMs are considered as one of the reasons for tumor radiation‐resistance in breast cancer patients. Preclinical and clinical data show the number of TAMs is correlated with poor prognosis in breast cancer patients. Several studies have investigated targeting TAMs as a therapeutic strategy for breast cancer patients. For example, Jones et al. has shown enhancement of non‐irradiated tumor regression and increased CD8^+^ T cells infiltration after combining radiotherapy with depleting recruited macrophages by anti‐CSF‐1 antibodies.^15^ Zhu et al. has also shown that the RIAE can be inhibited by depletion of macrophages in the 4T1 dual tumor mouse model.^29^ Nishiga et al. found that blockage of CD47 during radiotherapy can increase the phagocytosis ability of macrophages and activate the macrophage‐mediated abscopal effect in a SCLC mouse model, and this was T cell independent.^16^ All of these studies show that TAMs play an important role in radiation‐induced resistance and the enhancement of abscopal effect. These prior findings provided the motivation for our current study to determine if combining the TAM modulating agent, ferumoxytol, with radiotherapy might improve treatment efficacy.

In our study, radiotherapy (8 Gy ×3) alone or combined with ferumoxytol did not enhance abscopal tumor regression. This is in contrast to Zhu et al., where hypo‐fractionated radiotherapy led to enhanced abscopal effect and reduced lung metastases in the 4T1 dual tumor mouse model.^29^ A main difference between Zhu et al. and the current study was placement of the flank tumor, which for Zhu et al. occurred in another mammary fat pad 2‐day post‐primary tumor implantation. Another difference would be the radiation dose rate applied. While we used 1.75 Gy/min in our study, Zhu et al. used 2.40 Gy/min in their study. Although both dose rates range in the conventional radiotherapy zone,^30^ several studies showed that higher dose rates could produce more double‐strand breaks (DSBs) in the cytoplasm of cancer cells,^31^, ^32^, ^33^ leading to desirable immunogenic cell death.^1^, ^2^ These findings suggest that the abscopal tumor implantation procedure and the radiation dose and dose rate could influence the observation of abscopal effect. Prior work suggested the optimal radiation dose for stimulating anti‐tumor immune effects in our preclinical model was 8 Gy × 3, and that is why it was used here.^2^, ^29^, ^34^, ^35^ However, the optimal radiation dose for eliciting anti‐tumor immune effects may be different and warrants further study.^36^, ^37^

In our study, around 50% of the cells within the tumor mass were leukocytes, and around 15% of the cells were macrophages. In addition, we found the FMX group tumor size was negatively correlated with the population of CD206^−^CD80^+^ M1 macrophages, suggesting larger tumors have fewer M1 macrophages. Whereas in the Comb group, tumor size was positively correlated with CD206^+^CD80^−^ M2 macrophages, suggesting larger tumors have greater numbers of M2 macrophages. These disparate findings indicate the macrophage phenotype (M1 or M2) most responsible for driving tumor progression, may be dependent on the type of treatment. Nonetheless, high levels of CD206^−^CD80^+^ M1 macrophages in both primary and flank tumors were observed among all treatment groups, which was contrary to expectations and indicates this 4T1 model was associated with inflammation.^38^, ^39^ This suggests the TME was rich in iron, released from inflammatory cells, which can lead to T lymphocyte anergy and death^40^ as well as immunotolerance in antigen‐presenting cells (APCs).^41^ Our present strategy was to give ferumoxytol iron to polarize the M2 macrophage into the M1 phenotype, however, most of the macrophages in the tumors were already M1 polarized, even in the Ctrl group. Thus, providing the excess ferumoxytol iron could have negative effects in this highly inflammatory model. It could be the reason, in our study, there were a large amount of CD3^+^ T cells present without CD4 or CD8 expression^42^ in the tumors (data not shown) and the APCs showed low activity, that is MHC class II downregulation. This kind of tumor could be treated with iron chelating agents, such as deferasirox, and ciclopirox olamine, or antioxidant, such as ascorbate, to reduce the imbalance of oxidative stress. This iron chelation or antioxidant, combined with radiotherapy and ICIs, that is cytotoxic T‐lymphocyte associated protein 4 (CTLA‐4), may be a more adequate combination treatment for enhancing RIAE in our animal mode and is an area of future work.

The desirable immunomodulating effect of ferumoxytol is to polarize the M2 TAMs into M1 TAMs in the TME.^23^ Although some research observed ferumoxytol repolarized TAMs from M2 to M1, it also has been reported that ferumoxytol could reprogram macrophages from M1 to M2 or lead to loss of both phenotypes.^43^ In our study, the CD206^−^CD80^+^ M1 macrophages was the predominant population in all tumors, whereas the second greatest population was CD206^+^CD80^+^ macrophages, which can show “M1‐like” or overlapping M1/M2 features.^38^, ^39^ In the FMX group, contrary to expectations, the CD206^+^CD80^−^ M2 population was significantly increased relative to the Ctrl group. Whereas the CD80 and CD206 expression on the total macrophage population was similar in both tumors among all groups. These data suggested the desirable immunomodulating effect of ferumoxytol was not observed in our tumor model since there were no significant changes in the M1 macrophage levels across treatment groups. Furthermore, the MHC class II downregulation on the macrophages in the RT, FMX, and Comb groups suggested the macrophages lost their capability for antigen presentation, even though the costimulatory molecule, CD80, remained at a similar expression level. This suggests the radiotherapy and ferumoxytol treatments may actually be enhancing immune suppression within the TME. The significant increase in MHC class II upregulation CD206^−^CD80^−^ macrophages in both tumors in the RT, FMX and Comb groups, provides further support of severe inflammation in tumors after treatment. The percentage of MHC class II^+^ tumor cells showed similar expression level in tumors among all groups. This MHC class II expression on the tumors without the presence of costimulatory molecule, CD80, could lead to CD4^+^ T cell tolerance through anergy and the induction of regulatory T cells (Treg) expansion,^44^ resulting in the immunosuppressive TME and immune escape of the tumors.

HMGB1 is a double‐edged sword which has been linked to anti‐tumor and pro‐tumor responses after radiotherapy.^45^ Indeed, radiation‐induced HMGB1 released from cancer cells has shown to activate the systemic immune response in several tumor types, including the 4T1 mammary tumor.^29^ However, some studies suggested that reduction of HMGB1 in the plasma could inhibit the 4T1 tumor growth by remodeling the TME.^46^ Based on our results, the high HMGB1 levels in our animal model are likely supportive of a pro‐tumor response instead of an anti‐tumor response. The level of HMGB1 contributed by the radiation‐induced HMGB1 release in RT and Comb group was surprisingly less than expected. This may be because radiation induces formation of the oxidized‐HMGB1, which is less sensitive to recognition by anti‐HMGB1 antibodies in the ELISA.^46^ This may also explain why the HMGB1 level in the FMX group is lower than the Ctrl group, since the ferumoxytol can produce reactive oxygen species (ROS) via the Fenton reaction to oxidize the HMGB1. This oxidized‐HMGB1 can abrogate the immunostimulatory activity and pro‐inflammatory signals. Hubert et al. showed that the oxidized‐HMGB1 induces DCs' tolerogenicity, which leads to an immunosuppressive TME.^46^ Moreover, the common markers of tolerogenic DCs are CD11c and low expression of MHC class II,^47^ which is also observed in our results. Overall, this may contribute to the failure of the abscopal effect in our study because either RT and/or FMX could generate too much ROS and oxidative stress in the tumors.

Non‐invasive visualization of LN metastasis is crucial for providing information on therapeutic strategies for different stages of tumor progression.^48^ Macrophages have shown tumor‐targeting capability as well as metastasis‐regulating ability in several studies.^49^, ^50^ In addition, prior studies have observed the expression level of MHC class II on the mammary tumor cells in tumor‐draining lymph nodes (TDLNs) was positively correlated with the progression of the metastasis in the LNs, by eliciting the CD4^+^ T cell tolerance and an immunosuppressive TME.^44^ In our study, we evaluated the macrophage population in the right (near the primary tumor) and left inguinal LNs (near the abscopal tumor) by ferumoxytol MRI. Both inguinal lymph nodes showed T2* values significantly decreased by the 4th MRI scan, suggesting the ferumoxytol‐engulfed macrophages were accumulated and retained in both inguinal LNs. A significant positive correlation between the %T2* change in left inguinal LNs and lung metastasis nodules was observed in the Comb group. This might suggest a relationship between the macrophage infiltration at the abscopal side of the inguinal LNs and an induction of lung metastases after radiotherapy. The investigation of macrophage phenotype in the inguinal LNs by immunohistochemistry is needed for further verification.

Our data showed that the MRI %T2* changes were negatively correlated with CD206^−^CD80^+^ M1 macrophage levels in the FMX primary and flank tumors. This is in agreement with a previous study, which observed ferumoxytol MRI may selectively assess the M1 macrophage phenotype.^19^ Mohanty et al. demonstrated that CD47 blockage in a human osteosarcoma model can increase the CD80^+^ M1 macrophage population, enhance the phagocytosis of ferumoxytol, and significantly shorten the MRI‐measured T2* values.^51^ This is also consistent with our results in the FMX treatment group: the more the CD206^−^CD80^+^ M1 in the tumor, the more ferumoxytol could be retained, and the lower the %T2* change. This suggests the %T2* change in tumors can represent the CD206^−^CD80^+^ M1 macrophage population. Interestingly, we found the CD206^+^CD80^−^ M2 macrophage population was not correlated with the %T2* change. This suggests ferumoxytol MRI provides a selective measure of M1‐like macrophages. However, after radiation treatment, this correlation between MRI T2* and M1 macrophage levels was no longer significant, suggesting radiotherapy may diminish the capabilities of ferumoxytol MRI for measuring macrophages. Thus, utilization of ferumoxytol MRI may be best applied prior to the start of therapy, for patient stratification or prognostication.

Although ferumoxytol did not enhance the RIAE in our animal model, it has in other studies showed remarkable immunomodulating effects.^23^, ^24^, ^52^ The M2‐to‐M1 repolarizing effect of ferumoxytol is well characterized by several studies, which showed tumor growth inhibition and prevention of metastasis formation.^23^ However, these effects are likely dependent on the tumor types, the iron homeostasis of the tumors,^53^ and the concentration of ferumoxytol within tumor tissue and TAMs (e.g. 2.73 mg/mL administered in one reference).^23^ A relatively high concentration of ferumoxytol may be required for anti‐tumoral effects and the lack of sufficient ferumoxytol in the tumor may be one of the reasons our study differs from prior studies. To achieve higher concentrations of ferumoxytol within the tumor, future studies may attempt local delivery via surgery, interventional procedures, or microinjections at the tumor sites. These administration routes could potentially expose TAMs to an adequate amount of ferumoxytol, leading to greater M1 macrophage polarization and a greater likelihood for synergy with radiation therapy. Regardless, our results do support ferumoxytol MRI being used to measure M1 TAMs prior to treatment. This non‐invasive MRI evaluation of M1 TAMs could be used to adjust upcoming treatments based on TAM levels. This has great translational relevance as many new immunotherapies undergoing clinical trials target macrophages and there are no existing biomarkers that can reliably assess macrophage populations. Ferumoxytol MRI could be utilized to inform which patients have high levels of M1 TAMs and therefore be good candidates for participating in trials with TAM targeted immunotherapies. Furthermore, the pharmacodynamic effects of new TAM targeted therapies on altering M1 macrophage levels could also be assessed longitudinally with ferumoxytol MRI. Identifying which drugs successfully alter M1 macrophage levels would provide valuable go‐no‐go decisions on whether a new drug should move on to large clinical trials. This could also be worthwhile considering the emergence of adaptive radiation therapy (ART), the MR‐LINAC, and other radiotherapy‐immunotherapy combinations. Regardless, the translational relevance of a validated non‐invasive biomarker TAMs would be significant and add value to ongoing clinical studies.

## 5 CONCLUSION

In conclusion, we found that ferumoxytol combined with radiotherapy did not enhance RIAEs or improve the TME in our mammary cancer model. In addition, we observed no significant differences in immune cell populations across tumor sites, including primary orthotopic mammary tumors or flank tumors. Although ferumoxytol did not enhance abscopal effects, it did show potential for assessing M1 TAMs non‐invasively with MRI. Given ferumoxytol is already FDA‐approved, it could be translated relatively quickly into clinical trials to verify these MRI findings in human patients.

## CONFLICT OF INTEREST STATEMENT

The authors declare no conflict of interest.

## Supporting information



Supporting Information

## Data Availability

The data will be made available upon reasonable request.
