# Supplementary material for: Evaluating the theranostic potential of ferumoxytol when combined with radiotherapy in a mammary dual tumor mouse model
Source: Med Phys. 2025 May 21;52(7):e17888. doi: 10.1002/mp.17888 (PMC12257999; doi:10.1002/mp.17888)
Supplement: Supplementary file 1 — Supporting Information [file MP-52-0-s001.pdf]

Supplementary document:

Evaluating the theranostic potential of ferumoxytol when combined with radiotherapy in a mammary dual tumor mouse model

Deng-Yuan Chang[1], Joseph P. Speth[1] and Matthew L. Scarpelli[1*]

[1]School of Health Sciences, Purdue University, 550 W Stadium Ave, West Lafayette, IN 47907, USA

*Corresponding Authors: Matthew L. Scarpelli - orcid.org/0000-0003-0735-168;
Email: mscarpel@purdue.edu

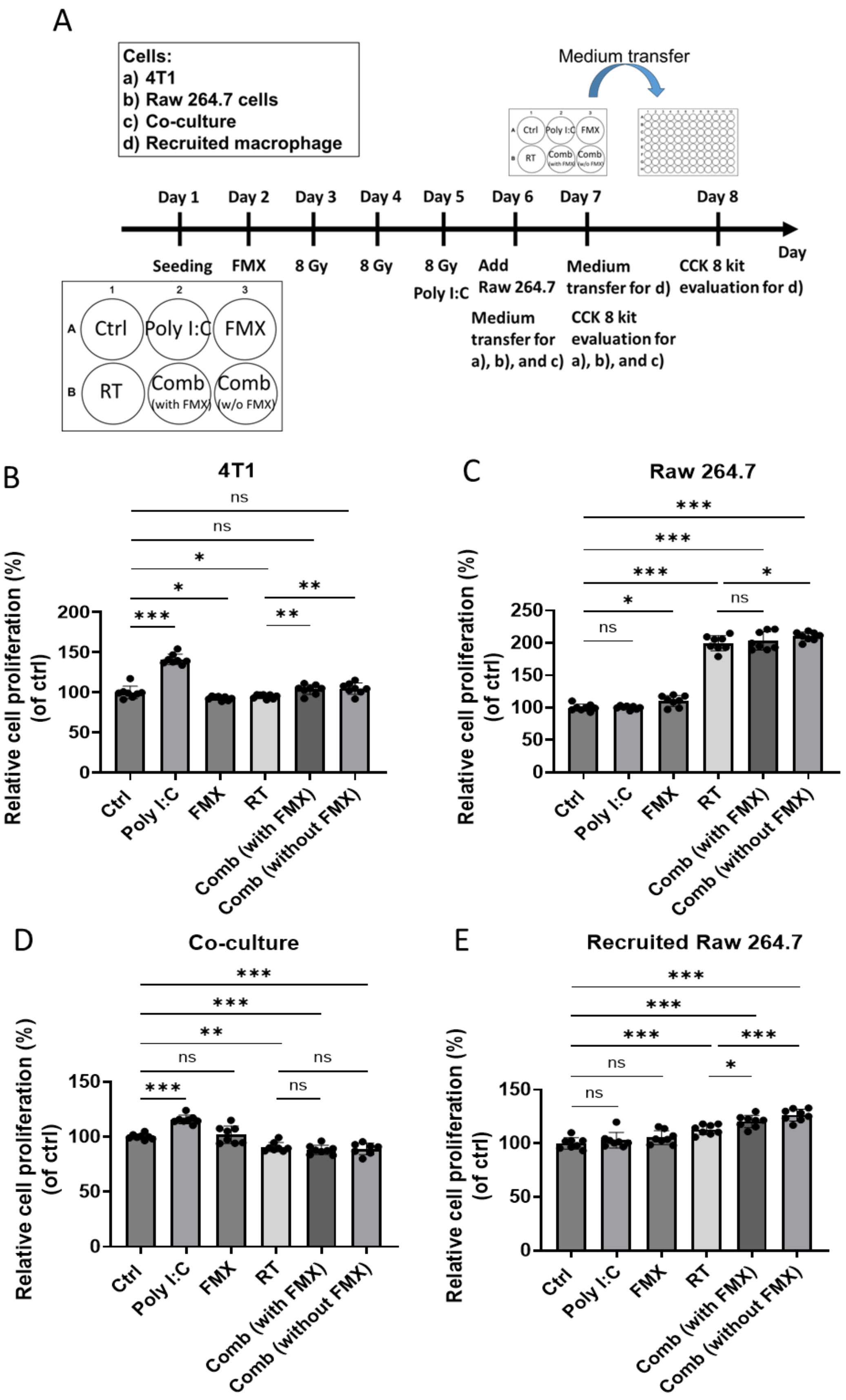
A
Cells:
a) 4T1
b) Raw 264.7 cells
c) Co-culture
d) Recruited macrophage
Medium transfer
Day 1
Day 2
Day 3
Day 4
Day 5
Day 6
Day 7
Day 8
Day
Seeding
FMX
8 Gy
8 Gy
8 Gy
Poly I:C
Add
Raw 264.7
Medium
transfer for
a), b), and c)
Medium
transfer for d)
CCK 8 kit
evaluation for
a), b), and c)
CCK 8 kit
evaluation for d)
Ctrl
Poly I:C
FMX
RT
Comb
(with FMX)
Comb
(w/o FMX)
B
4T1
C
Raw 264.7
D
Co-culture
E
Recruited Raw 264.7
Relative cell proliferation (%)
(of ctrl)
Ctrl
Poly I:C
FMX
RT
Comb (with FMX)
Comb (without FMX)

Fig. S1. The in vitro bystander effect was assessed by combining Ferumoxytol with RT in the 4T1, Raw 264.7, and co-culture system. (A) Schematics depicting the therapeutic schedule for RT and ferumoxytol. The cells were treated with 100 μg/mL of Ferumoxytol, if needed, for 24h, and then cells were irradiated with 8 Gy in three successive days. The 4T1 tumor proliferation rate was analyzed by incubating with the (B) medium transferred from the 4T1 plate, (C) medium transferred from the Raw264.7 plate, (D) medium transferred from the co-culture plate, (E) medium transferred from the recruited Raw 264.7 plate. The tumor proliferation rate significantly decreased in the poly I:C group from the recruited Raw 264.7 plate and co-culture plate compared to the 4T1 plate (Fig. S5D and S5E). Especially the poly I:C group from the recruited Raw 264.7 plate, it showed no proliferation difference compared to its control, suggesting the recruited M1 macrophage phenotype would not promote tumor growth. Data are represented as mean ± SD from 1 experiment and are representative of technical replicates. These data were compared by unpaired Student's t test. *$P < 0.05$; **$P < 0.01$; ***$P < 0.001$; ****$P < 0.0001$, n.s. represents no significance. Ctrl, Control; RT, Radiotherapy; FMX, Ferumoxytol; Comb (with FMX), Combined ferumoxytol with radiotherapy; Comb (without FMX), The ferumoxytol media was removed prior to radiotherapy; Poly I:C, Polyinosinic:polycytidylic acid serving as a positive control for converting Raw 264.7 to M1 phenotype

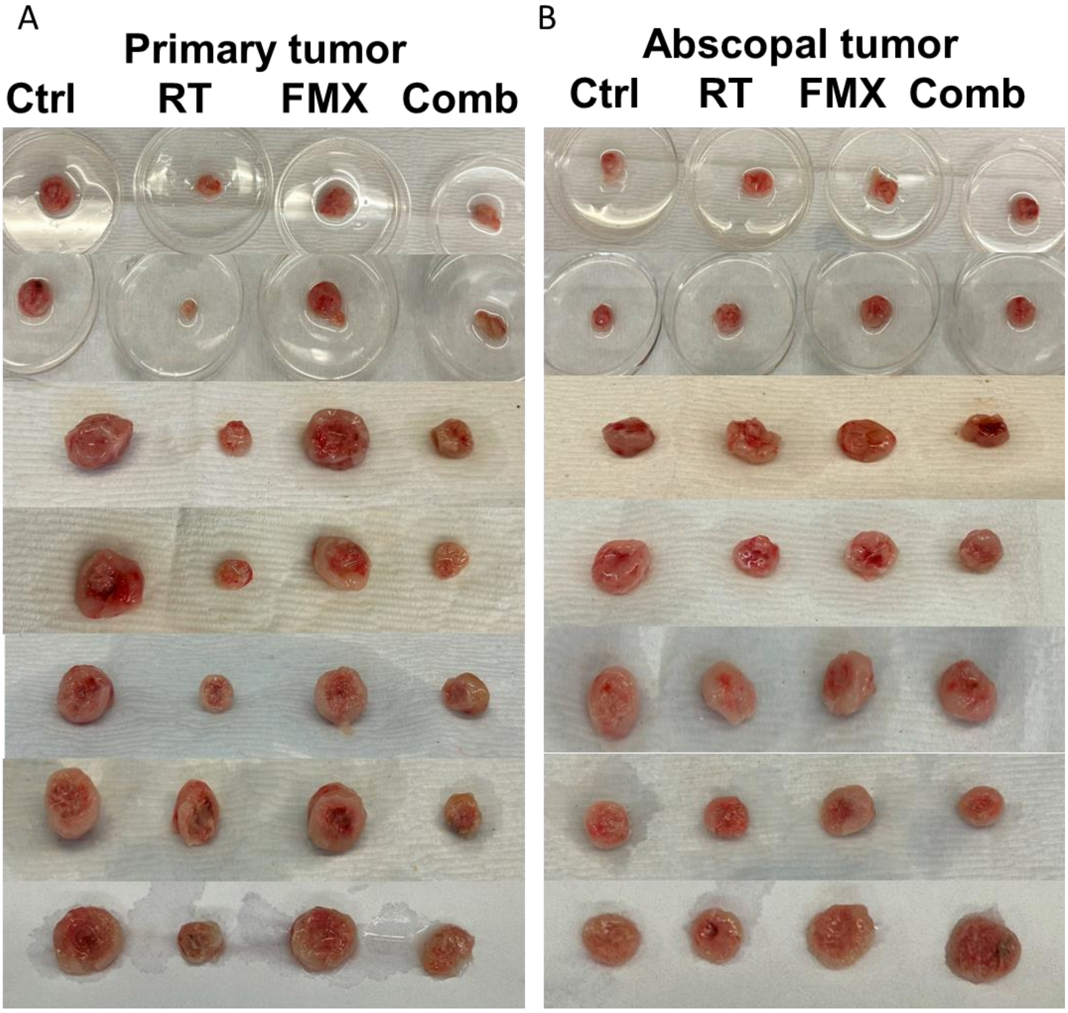

Fig. S2. The photograph of the (A) primary tumor and (B) secondary tumor after tumor harvest at 25-days post implantation.

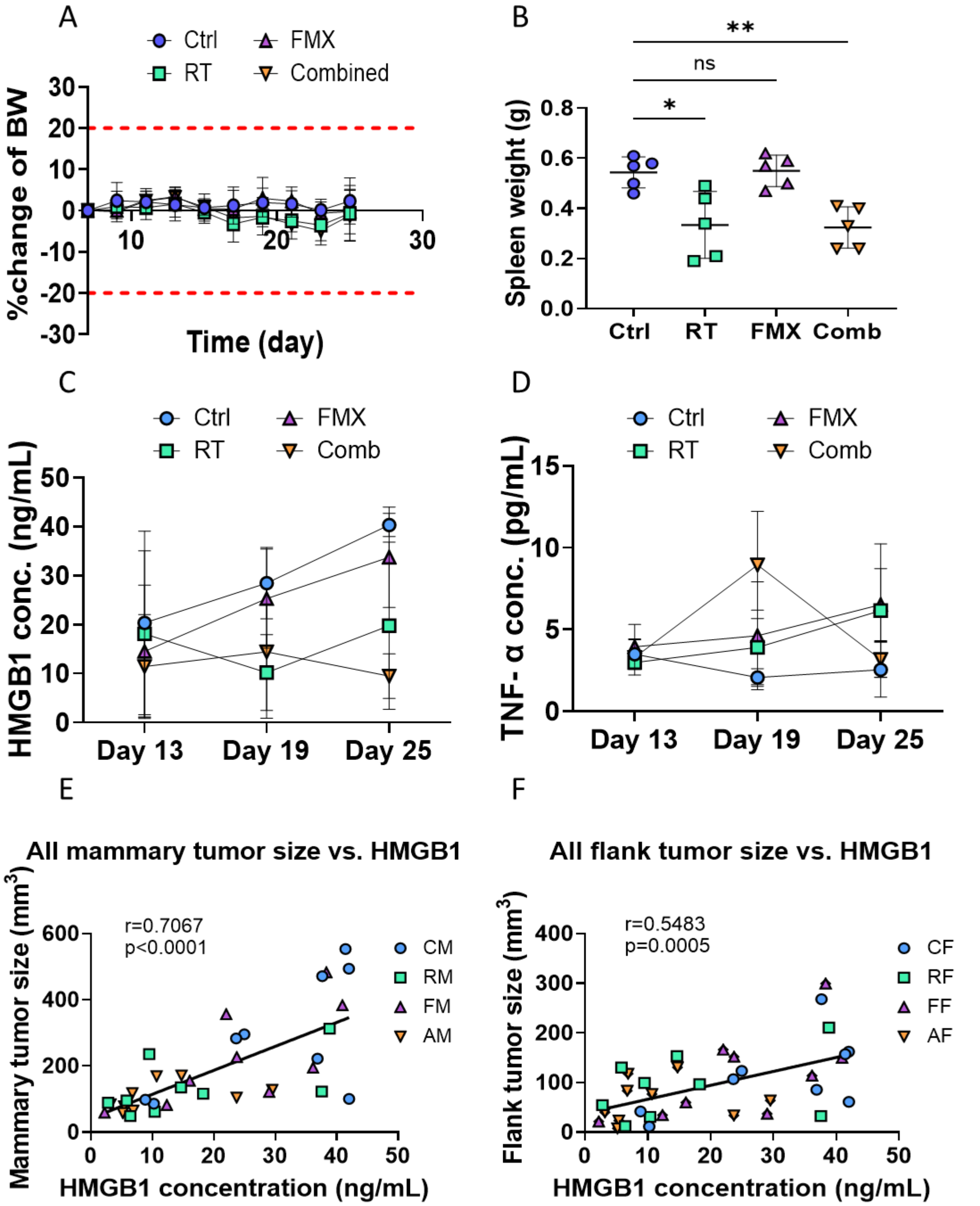

Fig. S3. Systemic toxicity and cytokines evaluation among all the treatments in the 4T1 dual tumor mouse model. A. The %change of the body weight of mouse in each group every other day at 7-day post-irradiation. (n = 7) B. The spleen weight of mice in each treatment group at the experiment endpoint (Day 25) (n = 5). The immunostimulatory DAMPs, (C) HMGB1, and the pro-inflammatory cytokines, (D) TNF-alpha, in the plasma during the experiment (n = 3). Pearson correlation for all the (E) mammary tumor sizes and (F) flank tumor sizes with the HMGB1 level at 3 given time points. Data are represented as mean ± SD. These data were compared by unpaired Student's t

test. *P < 0.05; **P < 0.01; ***P < 0.001; ****P < 0.0001, n.s. represents no significance. Ctrl, Control; RT, Radiotherapy; FMX, Ferumoxytol; Comb, Combined Ferumoxytol with Radiotherapy; HMGB1, High mobility group box 1; TNF-α, Tumor necrosis factor-alpha.

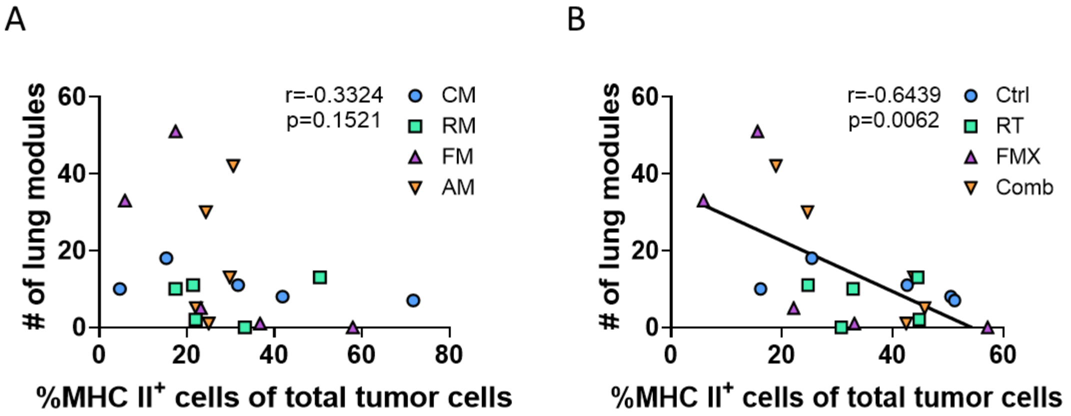

Fig. S4. A negative correlation between numbers of lung nodules and the %MHC class II^+ tumor cells. A.- B. A negative Pearson correlation was observed between numbers of lung metastases and %MHC class II^+ tumor cells of total tumor cells in all groups of (B) abscopal flank tumors but not in (A) primary tumors. MHC class II, Major histocompatibility complex class two; CM, Control mammary tumor; RM, Radiotherapy mammary tumor; FM, Ferumoxytol mammary tumor; AM, Combined treatment mammary tumor; CF, Control flank tumor; RF, Radiotherapy flank tumor; FF, Ferumoxytol flank tumor; AF, Combined treatment flank tumor.

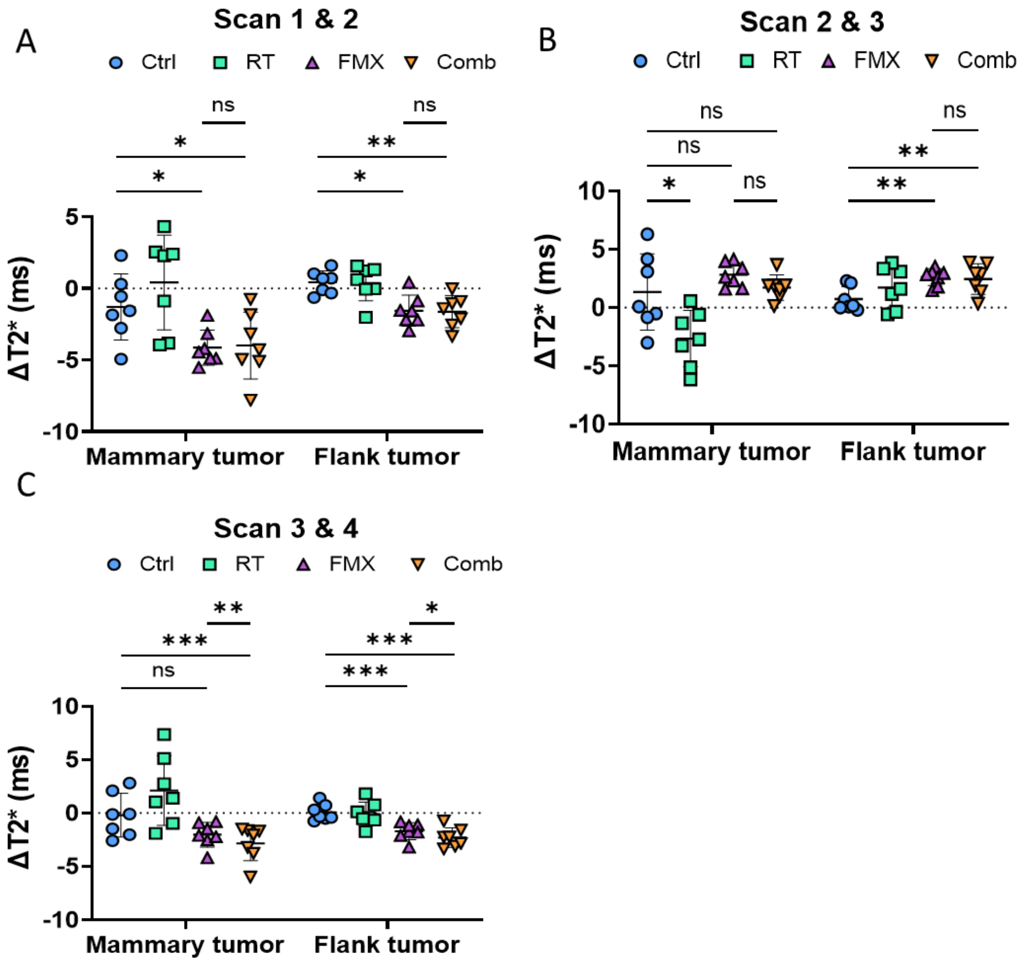

Fig. S5. The mean ΔT2* values of both tumors significantly decreased in FMX and Comb group, both before and after the radiotherapy. A. The ΔT2* values of mammary tumor and flank tumor from 1st and 2nd scans in both tumors among all groups at Day 14 & 15 (before the radiation treatment). B. The ΔT2* values of mammary tumor and flank tumor from 2nd and 3rd scans in both tumors among all groups at Day 15 & 23 (after the radiotherapy and before the next Ferumoxytol injection). C. The ΔT2* values of mammary tumor and flank tumor from 3rd and 4th scans in both tumors among all groups at Day 23 & 24 (6-day post-irradiation). A. – C. were compared by unpaired Student's t test with Mann-Whitney test. Data are represented as mean ± SD and are representative of 7 independent experiments. *$P < 0.05$; **$P < 0.01$; ***$P < 0.001$; ****$P < 0.0001$, n.s. represents no significance. Ctrl, Control; RT, Radiotherapy; FMX, Ferumoxytol; Comb, Combined Ferumoxytol with Radiotherapy.

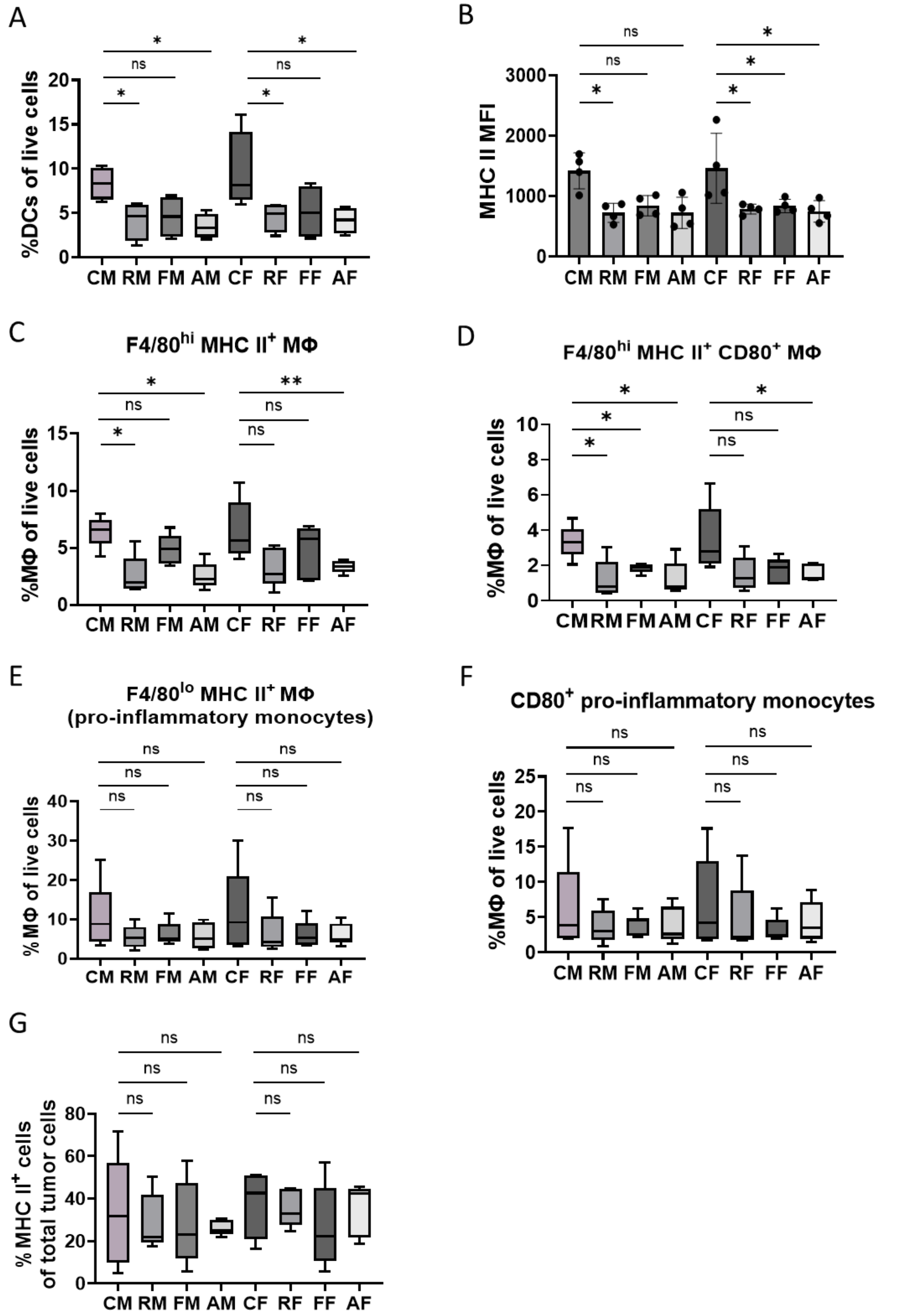

Figure S6. Decrease in DCs and F4/80^{hi} MHC II^{+} CD80^{+} MΦ were observed in Comb group tumors 8-days post irradiation, suggesting the loss of immunostimulatory population with antigen presenting capability after the combined treatment. (A) The

percentage of dendritic cells ($CD45^{+}$ $F4/80^{-}CD11c^{+}MHC\ II^{+}$ cells) of total live cells in each tumor among all groups. (B) The MFI of MHC II –FITC expressed on the DCs in all tumors. C.-G. The percentage of (C)$F4/80^{hi}$ MHC II^{+} macrophage, (D) $F4/80^{hi}$ MHC II^{+} $CD80^{+}$ macrophage, (E) $F4/80^{lo}$ MHC II^{+} macrophage, (F) $F4/80^{lo}$ MHC II^{+} $CD80^{+}$ macrophage, (G) MHC II expressed tumor cells ($CD45^{-}CD11b^{-}MHC\ II^{+}$ cells) of total tumor cells ($CD45^{-}CD11b^{-}$ cells) in each tumor among all groups. Data are represented as the ranges (min to max) of the population and are representative of 5 independent experiments (4 independent experiments for DC population). These data were compared by unpaired Student's t test with Mann-Whitney test. *$P < 0.05$; **$P < 0.01$; ***$P < 0.001$; ****$P < 0.0001$, n.s. represents no significance. CM, Control mammary tumor; RM, Radiotherapy mammary tumor; FM, Ferumoxytol mammary tumor; AM, Combined treatment mammary tumor; CF, Control flank tumor; RF, Radiotherapy flank tumor; FF, Ferumoxytol flank tumor; AF, Combined treatment flank tumor; DC, Dendritic cell; MΦ, Macrophage; MHC II, Major histocompatibility complex class two.

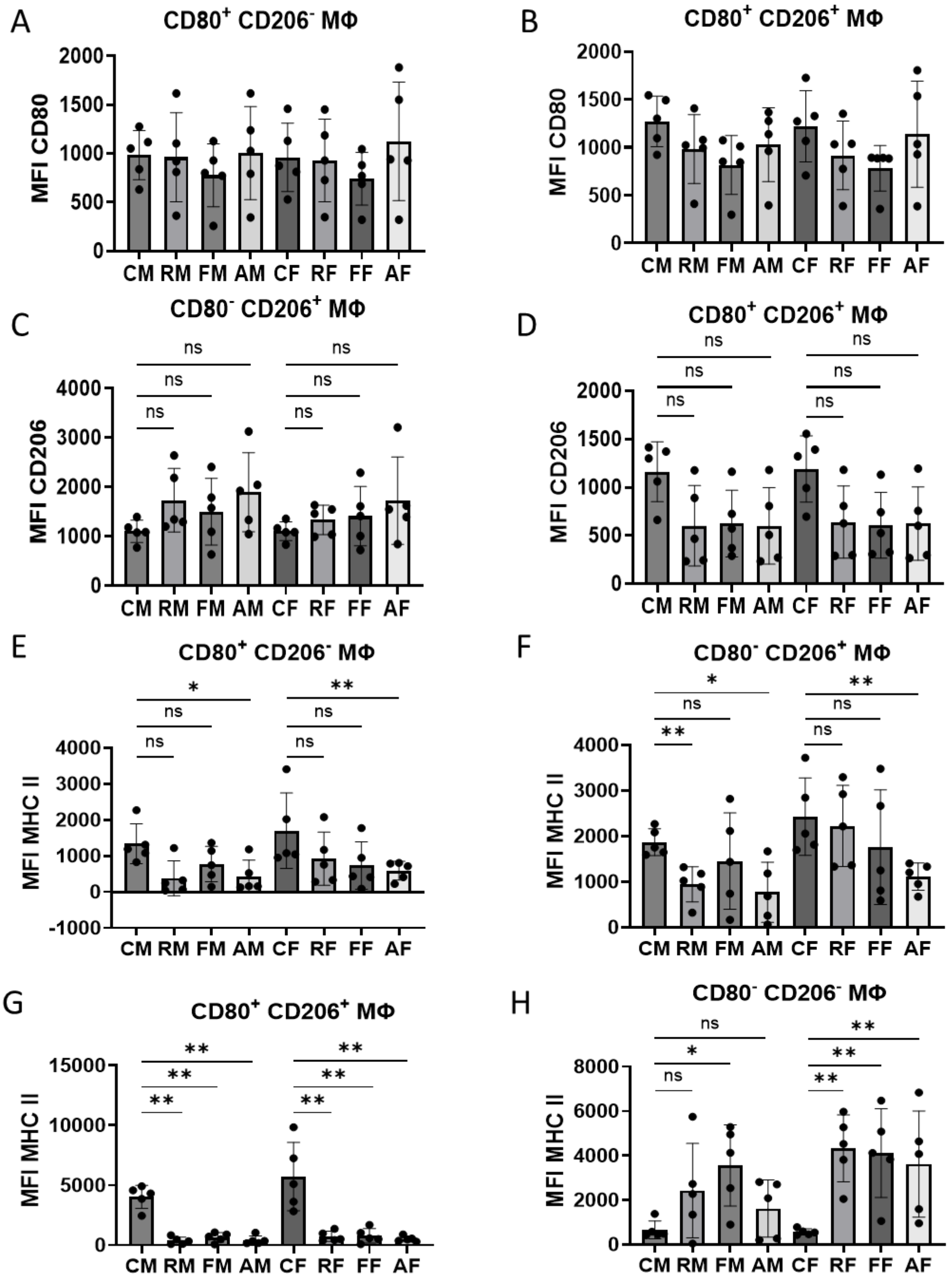

Fig. S7. Ferumoxytol combined with Radiotherapy downregulated the expression level of MHC class II in most of the macrophage phenotypes. The CD80 expression level was determined by MFI in (A) CD80$^+$ CD206$^-$ MΦ and (B) CD80$^+$ CD206$^+$ MΦ macrophage phenotypes among all groups. The CD206 expression level was determined by MFI in (C) CD80$^-$ CD206$^+$ MΦ and (D) CD80$^+$ CD206$^+$ MΦ macrophage phenotypes among all groups. The MHC class II level was determined by MFI in (E) CD80$^+$ CD206$^-$ MΦ, (F) CD80$^-$ CD206$^+$ MΦ, (G) CD80$^+$ CD206$^+$ MΦ, and (H) CD80$^-$ CD206$^-$ MΦ among all groups. These data were compared by unpaired

Student's t test with Mann-Whitney test. $*P < 0.05$; $**P < 0.01$; $***P < 0.001$; $****P < 0.0001$, n.s. represents no significance. CM, Control mammary tumor; RM, Radiotherapy mammary tumor; FM, Ferumoxytol mammary tumor; AM, Combined treatment mammary tumor; CF, Control flank tumor; RF, Radiotherapy flank tumor; FF, Ferumoxytol flank tumor; AF, Combined treatment flank tumor; DC, Dendritic cell; MHC class II, Major histocompatibility complex class two.

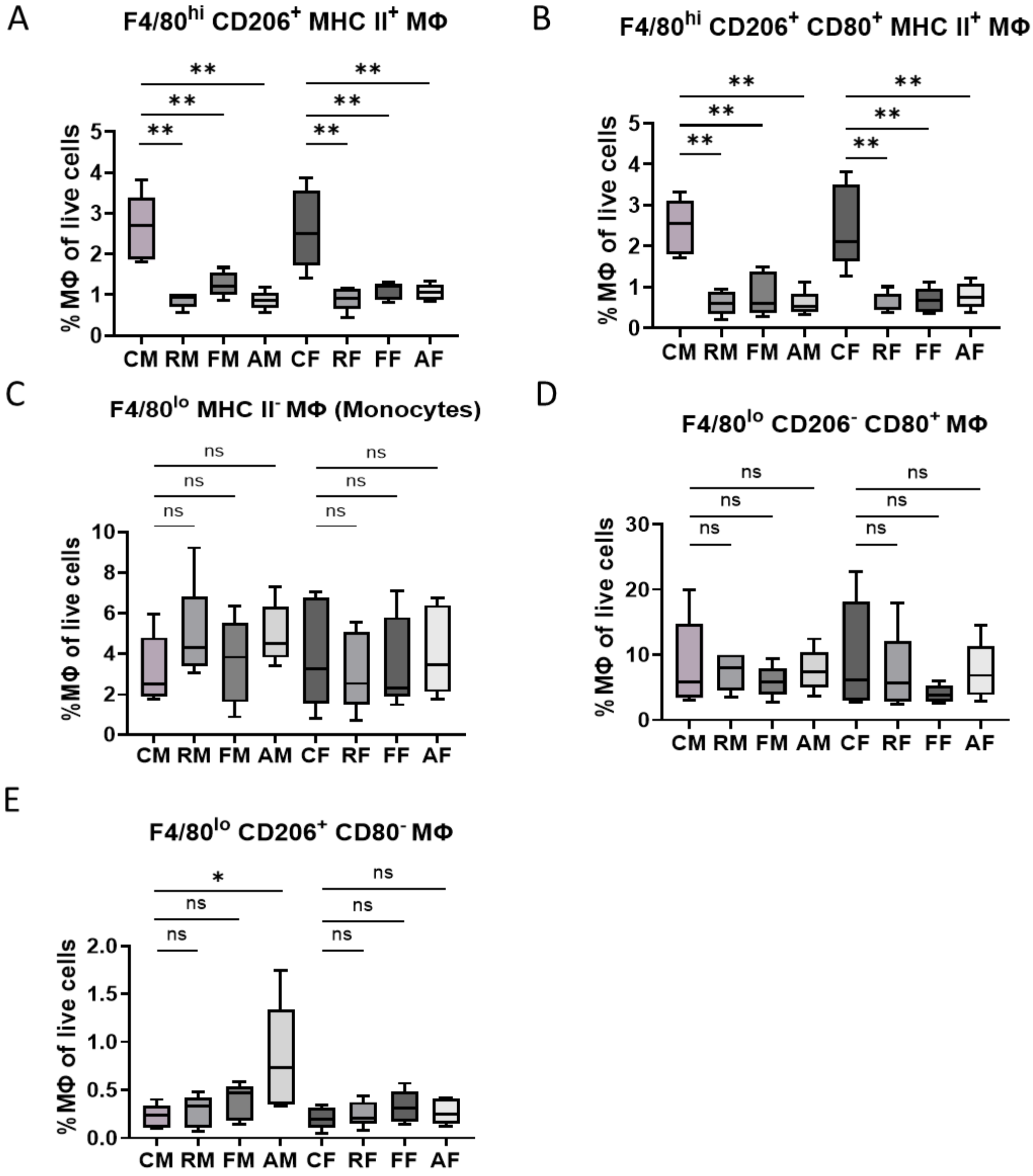

Fig. S8. A specific phenotype of resident macrophages, stromal type I macrophage, (F4/80^{hi} CD206^{+} MHC II^{+} macrophage) originating from the mammary fat pad decreased in both tumors in RT, FMX, and Comb group after 8-days post-irradiation. A. – E. The percentage of (A) F4/80^{hi} CD206^{+} MHC II^{+} MΦ, (B) F4/80^{hi} CD206^{+} CD80^{+} MHC II^{+} MΦ, and (C) F4/80^{lo} MHC II^{-} MΦ, (D) F4/80^{lo} CD206^{-} CD80^{+} MΦ, and (E) F4/80^{lo} CD206^{+} CD80^{-} MΦ of total live cells in both tumors among all group. Data are represented as the ranges (min to max) of the population and are representative of 5 independent experiments. These data were compared by unpaired Student's t test with Mann-Whitney test. *$P < 0.05$; **$P < 0.01$; ***$P < 0.001$; ****$P < 0.0001$, n.s. represents no significance. CM, Control mammary tumor; RM, Radiotherapy

mammary tumor; FM, Ferumoxytol mammary tumor; AM, Combined treatment mammary tumor; CF, Control flank tumor; RF, Radiotherapy flank tumor; FF, Ferumoxytol flank tumor; AF, Combined treatment flank tumor; MΦ, Macrophage; MHC class II, Major histocompatibility complex class two.

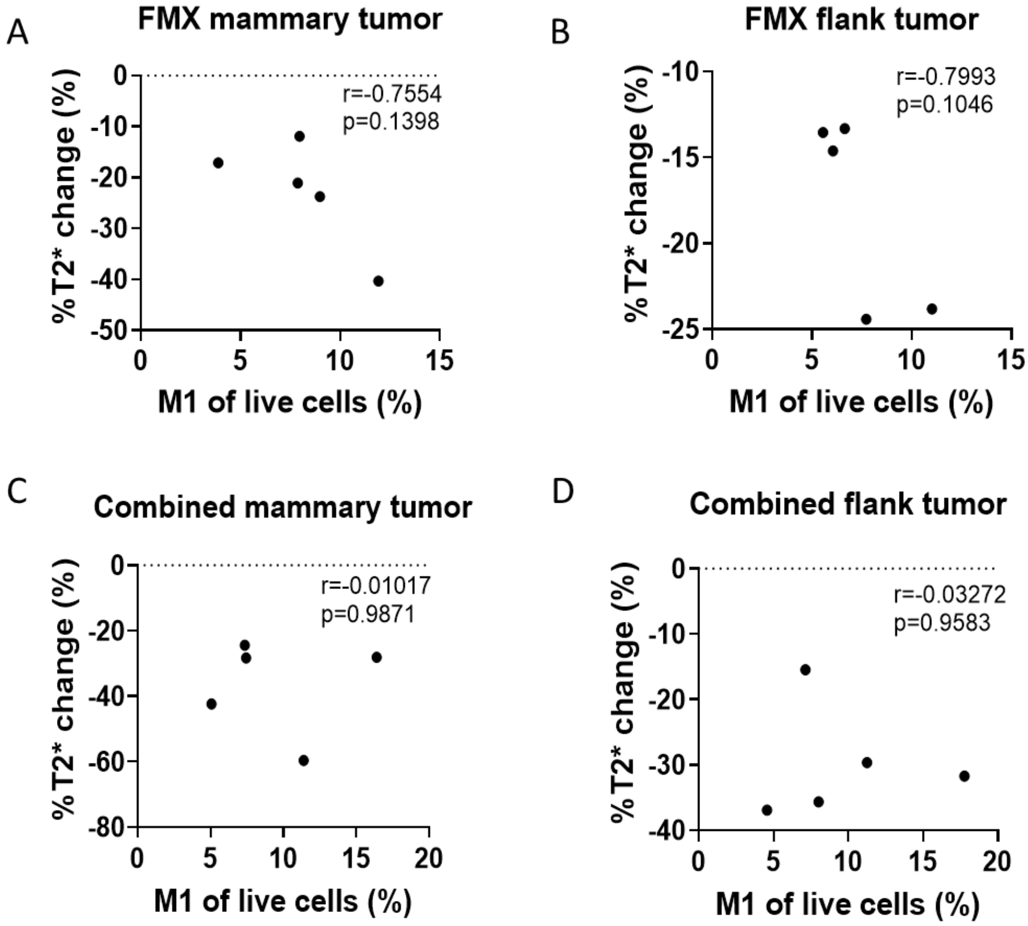

Fig. S9. The correlation between %T2* change and M1 $CD80^+$ $CD206^-$ macrophage population showed a negative correlation in flank and mammary tumors in the FMX group but not in the Comb group. A. – B. The Pearson correlation between %T2* change and $CD80^+$ $CD206^-$ macrophage population in (A) FMX mammary tumor or (B) FMX flank tumor. C. – D. The Pearson correlation between % T2* change and $CD80^+$ $CD206^-$ macrophage population in (C) Comb mammary tumor or (D) Comb flank tumor. FMX, Ferumoxytol; Comb, Combined Ferumoxytol with Radiotherapy; MΦ, Macrophage.

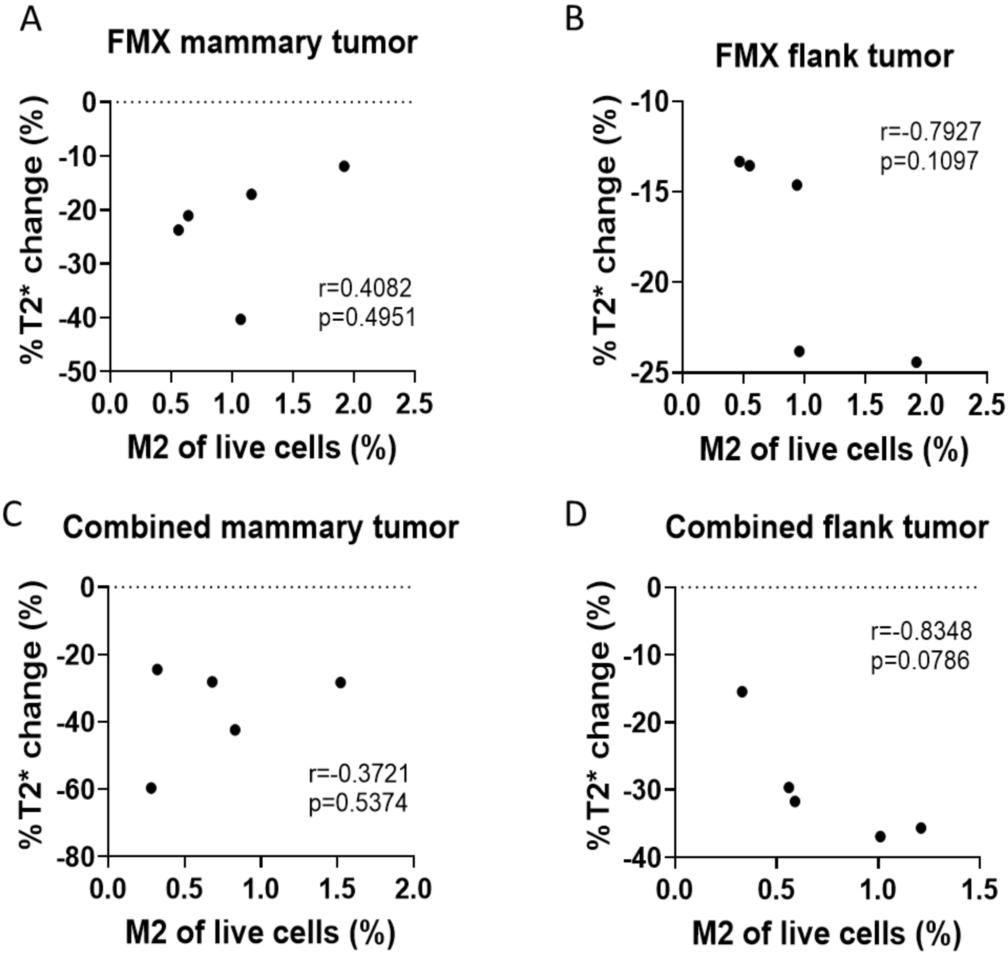

Fig. S10. No Pearson correlation was found between %M2 macrophages and %T2* change among all tumors in FMX and Comb group. A. – B. The Pearson correlation between % T2* change and $CD80^-$ $CD206^+$ macrophage population in (A) FMX mammary tumor or (B) FMX flank tumor. C. – D. The Pearson correlation between % T2* change and $CD80^-$ $CD206^+$ macrophage population in (C) Comb mammary tumor or (D) Comb flank tumor. FMX, Ferumoxytol; Comb, Combined Ferumoxytol with Radiotherapy; MΦ, Macrophage.

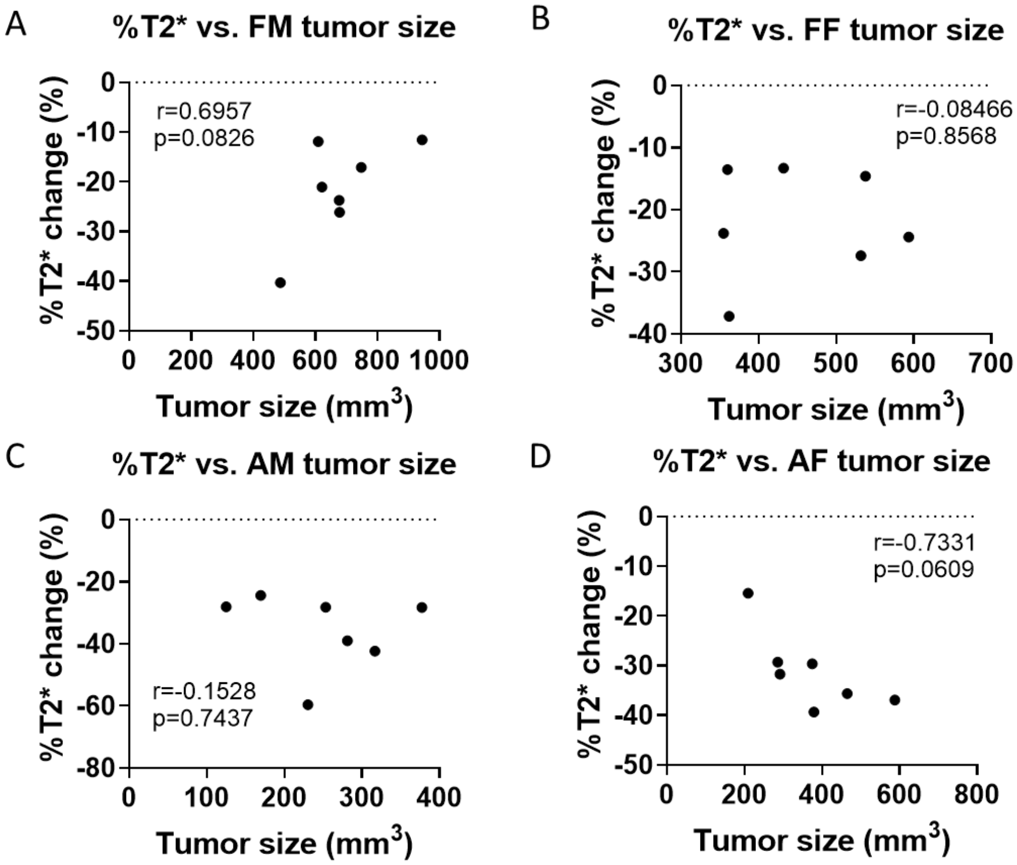

Fig. S11. No correlation was observed between %T2* change and the MRI tumor size measured 24 days post implantation. A.- D. The Pearson correlation between %T2* changes and MRI measured tumor size in (A) FMX mammary tumors, (B) FMX flank tumors, (C) Comb mammary tumors, and (D) Comb flank tumors. FM, Ferumoxytol mammary tumor; AM, Combined treatment mammary tumor; FF, Ferumoxytol flank tumor; AF, Combined treatment flank tumor.

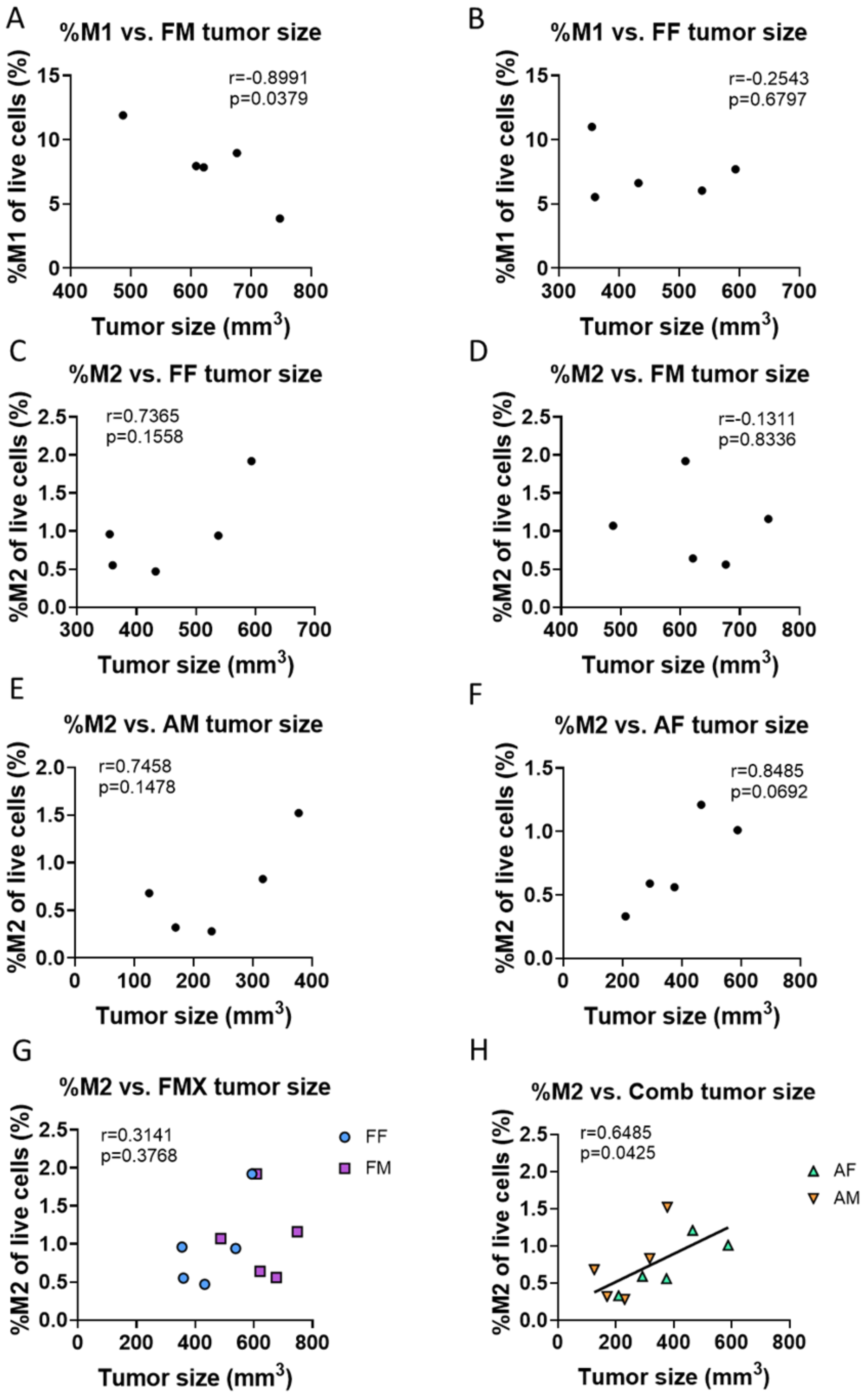

Fig. S12. A negative Pearson correlation was found between FMX group tumor size

with M1 macrophage population while a positive correlation was found between Comb group tumor size with M2 macrophage population. A.-B. The Pearson correlation between %M1 macrophages and tumor size in (A) FMX group mammary tumors, and (B) FMX group flank tumors. C.-H. The Pearson correlation between %M2 macrophages and tumor size in (C) FMX group mammary tumors, (D) FMX group flank tumors, (E) Comb group mammary tumors, (F) Comb group flank tumors, (G) the pooled FMX group tumors and (H) the pooled Comb group tumors. FM, Ferumoxytol mammary tumor; AM, Combined treatment mammary tumor; FF, Ferumoxytol flank tumor; AF, Combined treatment flank tumor.

Table S1. Mean transverse relaxation time (T2*) for mouse tissues.

Tissue	T2* (ms)
Mammary tumor	10.39 ± 1.90
Flank tumor	6.71 ± 1.27
Aorta	12.74 ± 2.64
Intra vena cava (IVC)	7.49 ± 1.33
Skeletal muscle	11.82 ± 2.95
Bone marrow	6.27 ± 0.81
Spleen	5.12 ± 1.06
Left LN	8.05 ± 1.59
Right LN	8.99 ± 1.69

The data was obtained from the baseline scan (1st scan) among all groups of mice (n= 28) and were represented as mean ± SD.

Table S2. Median transverse relaxation time (T2*) for mouse tissues.

Tissue	T2* (ms)
Mammary tumor	9.35 ± 1.85
Flank tumor	5.88 ± 1.53
Aorta	11.93 ± 2.80
Intra vena cava (IVC)	6.81 ± 1.33
Skeletal muscle	11.69 ± 3.16
Bone marrow	5.38 ± 0.94
Spleen	4.90 ± 1.12
Left LN	7.87 ± 1.60
Right LN	8.99 ± 1.92

The data was obtained from the baseline scan (1st scan) among all groups of mice (n= 28) and were represented as median ± interquartile range.
